# Predictive Modeling of Paracetamol Polymorphs: Linking Basis Set and Hamiltonian Effects to an Optimized Quasi-Harmonic Lattice Dynamics Workflow

**DOI:** 10.1021/acs.jctc.6c00750

**Published:** 2026-09-09

**Authors:** Huanyu Zhou, Giuseppe Mallia, Nicholas M. Harrison

**Affiliations:** † Department of Chemistry, 4615 Imperial College London, White City Campus, 80 Wood Lane, London W12 0BZ, United Kingdom; ‡ School of Aerospace Engineering and State Key Laboratory of Environment Characteristics and Effects for Near-space, 47833 Beijing Institute of Technology, Beijing 100081, China

## Abstract

Thermodynamic properties of molecular crystals are the cornerstone of a broad range of applications, spanning pharmaceutics, organic semiconductors, and many more. The reliable modeling of these properties is usually hindered by collective influences of delicate dispersion interactions and insufficient prior knowledge. Providing reasonable accuracy and minimal reliance on experimental data, lattice dynamics (LD) based on the quasi-harmonic approximation (QHA) and density functional theory (DFT) has been widely and successfully adopted for crystal structure prediction (CSP). However, for polymorphic molecular crystals, it remains challenging to accurately establish the relative stability among multiple polymorphs that are distinguished by subtle energy differences. Despite recent intense efforts to achieve calculations that can reproduce observations in well-characterized systems, a comprehensive study elucidating how predictions are governed by errors within the theoretical framework and details of implementation is lacking. This undermines confidence in the transferability and efficiency of QHA LD workflows proposed in the literature. The need for reliable and predictive calculations necessitates systematic investigation and disentangling of the influences from key parameters such as the choice of basis set and Hamiltonian, so the cost and precision of DFT-based QHA LD calculations can be rationally balanced in practical workflows. In this study, paracetamol polymorphs are adopted as the prototype system, as they are well-characterized and display representative chemical interactions, where covalent bonds, hydrogen bonds, and London dispersions are present with sufficient complexity. The intermixed sources of error underlying free-energy predictions are resolved by comparing the structural, vibrational, and thermochemical properties of paracetamol polymorphs with high-quality references. A hierarchy of atomic and plane-wave basis sets are adopted to highlight the significance of basis set completeness, and results with and without the inclusion of Fock exchange identify the particular importance of Hamiltonian for thermochemical properties. Furthermore, an optimal QHA LD workflow is proposed and tested utilizing these insights, which combines results from plane-wave basis set with semilocal functional and local, well-converged def2-TZVP basis set with hybrid functional to efficiently converge the numerical precision for QHA LD. While demonstrated in detail for paracetamol, conclusions of this study are also expected to be of relevance to a wide range of molecular crystals. These findings rigorously set the stage for affordable and reliable QHA LD workflow for molecular crystals, where fortuitous error cancellations are clearly distinguished from converged levels of theory, thereby clarifying the numerical precision attainable before explicit anharmonic and configurational effects are considered for complex organic systems.

## Graphical abstract

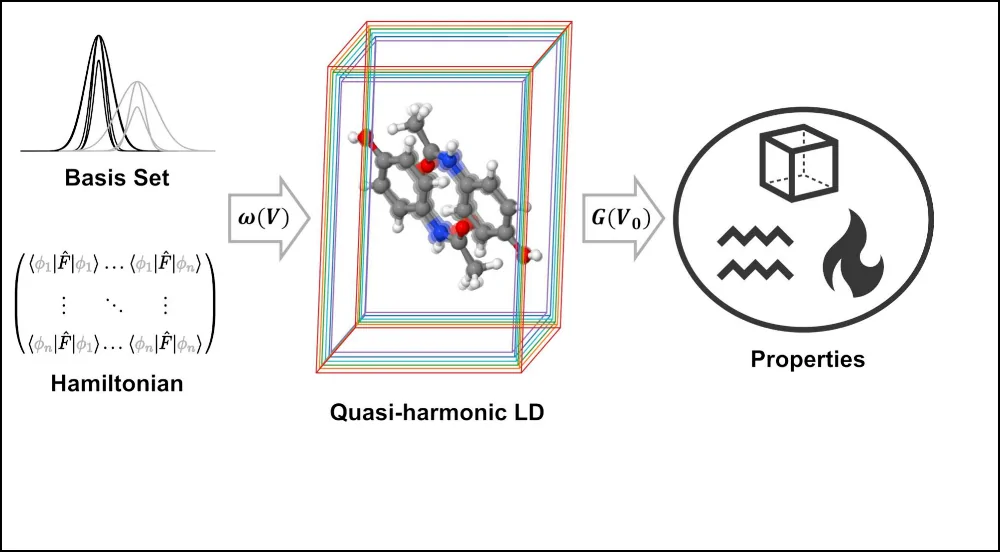

## Introduction

Molecular crystals are characterized by polymorphism, where identical molecules pack into distinct periodic motifs.^1^ In practical applications, the stability of these polymorphs is highly sensitive to inevitable fluctuations in environmental factors such as humidity, temperature, and pressure. This sensitivity presents a critical concern in process engineering, where obtaining a polymorph with desired structure, morphology, and properties can be vital for the efficiency of downstream processing.^2–6^ Furthermore, in the pharmaceutical industry, unwanted polymorphic transitions must be strictly avoided; such transitions can influence manufacturability, reactivity, and bioavailability, potentially compromising the effectiveness of the final product.^7^

Fundamentally, such vulnerability originates from the dominance of weak, noncovalent interactions within the lattice. The typical energy difference between molecular polymorphs is within 2 kJ mol^–1^, notably lower than the chemical accuracy of ∼4 kJ mol^–1^ accepted by many contemporary simulation methods as standard.^8–12^ As essential components of free energy, internal energy and vibrational entropy are correlated through the shared dependence on both geometry and lattice vibrations. The mixing of multiple potential sources of error means that, on many occasions, it is very difficult to unambiguously disentangle their individual influences on a prediction. Nonsystematic and contradictory influences can potentially yield fortuitous error cancellations in specific systems, which are hardly transferrable to other molecular crystals. Therefore, polymorph energy ranking remains challenging for crystal structure prediction (CSP), and these rankings can serve as a standard for validating theoretical models.

Density functional theory (DFT) has emerged as an appropriate tool for CSP, because of its predictive power, which typically yields better accuracy and transferability than preparameterized classical potentials. Several recent CSP workflows have incorporated DFT as an important complement to molecular dynamics (MD) based on classical potentials. Examples include screening the zeroth-order CSP landscape,^13^ correcting free energy of final candidates,^14,15^ and training machine-learning potentials.^15^ However, completely replacing classical potentials with DFT is so far computationally prohibitive for MD-based free energy methods, where, at least, each polymorph must be ergodically sampled with the costly ab initio MD method, despite recent efforts to avoid alchemical samplings that might further escalate time consumption.^16,17^ Alternatively, by approximating thermal effects as the excitation of harmonic phonon modes, lattice dynamics (LD) can consistently implement DFT throughout the CSP workflow at reasonable cost. In recent years, DFT-based LD has been successfully adopted to calculate the vibrational properties of a wide range of materials, including metals,^18–21^ minerals,^22–25^ metal–organic frameworks,^26^ and molecular crystals,^27–32^ suggesting its general applicability. In the latest CSP blind test, this method was adopted by four participating groups of ten who reported free energies, and overall good agreement with the references was achieved.^33^

Despite these achievements, unique difficulties are encountered for molecular crystals due to their peculiar physicochemical properties. The LD-based free-energy method neglects the high-order couplings between phonon modes (i.e., anharmonicity), as well as the interconverting lattice minima at finite temperatures. These effects become increasingly prominent for large and flexible molecules with the complexity of typical pharmaceutical compounds, as evidenced by a comparative study of MD-based and LD-based free-energy approaches.^34^ Besides, the DFT-derived potential energy surface (PES) plays a fundamental role, determining the accuracy of any free energy method that employs DFT. Its parametrization, such as basis set and Hamiltonian, is therefore another critical source of error. Basis set incompleteness error is an inherent drawback of any practically adopted basis set of finite size. For localized basis sets routinely adopted in computational chemistry studies, this incompleteness specifically manifests as the basis set superposition error (BSSE), which can overestimate intermolecular bindings by over 40% ^35,36^ and consequently leads to fictitious blue-shifts in predicted phonon frequencies.^37^ In addition, the commonly adopted semilocal DFT Hamiltonian fails to account accurately for long-range electron correlation, as exemplified by noncovalent intermolecular interactions, and short-range correlation of localized electrons. This necessitates the use of more advanced levels of theory, such as dispersion correction,^38,39^ nonlocal Fock exchange,^40^ or orbital-dependent correlation.^41–43^ Although the numerical precision of DFT-based LD can be consistently improved by ascending the hierarchy of underlying DFT models, the increased computational cost could limit its use for larger molecules (more than 20 non-hydrogen atoms) and complex unit cells (more than one symmetrically irreducible molecule).^44^ Achieving a rational parametrization of basis set and Hamiltonian is therefore crucial to balance the accuracy and efficiency of such calculations for use in practical CSP workflows.

In our previous investigation of LD based on harmonic approximation (HA),^45^ the importance of achieving basis set completeness has been stressed as a prerequisite for evaluating the improvements offered by the inclusion of Fock exchange. However, non-negligible deviations are seen to persist between the fixed-geometry predictions inherent for HA and variable-temperature measurements. These discrepancies escalate at higher temperatures, suggesting a fundamental failure of the HA. This underscores the need to account for thermal expansion through the quasi-harmonic approximation (QHA), where thermal effects on geometry are incorporated via establishing the volume-dependence of phonon frequencies.

In the current work, the widely used pharmaceutical, paracetamol, is studied as a representative system to systematically document the influences of basis set and Hamiltonian that might undermine the predictive polymorph energy ranking with DFT-based QHA LD. Polymorphs of paracetamol—namely, forms I (FI) and II (FII)—are uniquely suited, since they capture the complex interplay of covalent bonds, hydrogen bonds, London dispersions and π–π stacking, which are characteristic for practical molecular materials. By evaluating a hierarchy of atomic basis sets against converged plane-wave calculations, we demonstrate how to efficiently obtain numerically converged QHA LD predictions for both structural and vibrational properties. We further illustrate that the inclusion of Fock exchange is paramount for accurate thermochemical predictions. The analysis leads to a robust composite strategy that employs plane-wave basis sets with (semi)­local functionals to determine equilibrium volumes at finite temperatures, followed by the refinement using hybrid-DFT and atomic basis sets with comparable precision for thermochemistry and other properties where the electron structure has more prominent contributions. This approach balances accuracy and computational cost, providing a transferable framework for the predictive modeling of complex molecular crystals.

## Methodology

### Basis Set and Hamiltonian

All-electron Gaussian basis sets, including the double-ζ 6–31G** (from ref 46) and def2-SVP,^47,48^ and the triple-ζ pob-TZVP^49^ and def2-TZVP,^47,48^ are considered in this work to systematically document the influences of basis set cardinality and diffuseness. To emphasize the localized feature of Gaussian basis sets, in this work, they are referred to as atomic basis sets, in contrast to the delocalized plane-wave basis set. The semiempirical geometrical counterpoise (gCP) method with recommended parametrization is used for all atomic basis sets to correct BSSE.^50,51^ This method depends only on lattice geometry and hence is more efficient for large and periodic systems compared to the conventional counterpoise method. The plane-wave basis set is adopted as a BSSE-free reference, whose kinetic energy cutoff for electron wave function is set to 100 Ry.

The generalized-gradient-approximated Perdew–Burke–Ernzerhof (GGA-PBE) functional^52^ and its hybrid variant with 25% Fock exchange, PBE0,^53^ are used to compare results based on DFT and Hartree–Fock (HF) Hamiltonians. Only GGA-PBE is adopted for plane-wave calculations due to the prohibitive computational load of calculating Fock exchange. To account for London dispersion interactions, the a posteriori D3 method is adopted with three-body term and Becke–Johnson damping (D3-BJ),^54,55^ which achieves a balanced performance between cost and efficiency, as suggested by previous studies on organic molecular crystals.^38,39,56,57^ Since D3-BJ is adopted consistently here, for simplicity, “PBE” and “PBE0” respectively stand for the conventional annotations “PBE-D3­(BJ)” and “PBE0-D3­(BJ)” in the following text.

### Free Energy

Within the theoretical framework of QHA, the Helmholtz free energy *F* of a solid crystal is a function of temperature *T* and lattice volume *V*:

1 $$F\left(V,T\right)=U_{\mathrm{DFT}}\left(V\right)+F_{\mathrm{vib}}\left(V,T\right)+F_{\mathrm{el}}\left(V,T\right)$$

where *U*_DFT_ is the static internal energy obtained by the self-consistent field (SCF) method. *F*_el_ is the free energy of electron excitation at finite temperatures, which is neglected in this work considering the wide band gap of targeted systems. *F*_vib_ is the vibrational free energy obtained by LD, which is derived from angular phonon frequencies ω:

2 $$F_{\mathrm{vib}}\left(V,T\right)=\frac{1}{n}\overset{n,3N}{\underset{q,i}{\sum }}\frac{1}{2}\hbar \omega_{q,i}\left(V,T\right)+\frac{1}{n}\overset{n,3N}{\underset{q,i}{\sum }}k_{B}T\ln\left[1-\exp\left(-\frac{\hbar \omega_{q,i}\left(V,T\right)}{k_{B}T}\right)\right]$$

where *n* and *N* are, respectively, the number of ***q*** vectors in reciprocal space and the number of atoms in unit cell. The summation is taken over 3*N* nontranslational normal modes (3*N* – 3 at the Γ-point) sampled on the equally spaced and equally weighted ***q*** mesh. *ℏ* denotes the reduced Planck constant. *k*_B_ denotes the Boltzmann constant.

### Volume Dependence

To circumvent computationally prohibitive free-energy minimization, *U*_DFT_ and ω are sampled on a set of volume points around the fully optimized athermal equilibrium volume. The lattice and the atomic coordinates of each expanded or contracted geometry are relaxed subject to the corresponding volume, so thermal effects on the cell and molecular configuration can be indirectly accounted for with affordable cost. In this context, the geometry is displaced and optimized with constraints on the athermal PES, rather than the free energy PES.

The volume dependence of static internal energy *U*_DFT_ is expressed in the isothermal third-order Birch–Murnaghan equation of state (EoS):^58^

3 $$U_{\mathrm{DFT}}\left(V\right)=U_{0}+\frac{9V_{0}B_{0}}{16}\left\{{\left[{\left(\frac{V_{0}}{V}\right)}^{2/3}-1\right]}^{3}B_{0}^{\prime }+{\left[{\left(\frac{V_{0}}{V}\right)}^{2/3}-1\right]}^{2}\left[6-4{\left(\frac{V_{0}}{V}\right)}^{2/3}\right]\right\}$$

where *U*_0_, *V*_0_, *B*_0_ and $B_{0}^{\prime }$ are the equilibrium internal energy, volume, bulk modulus, and the first derivative of the bulk modulus at zero pressure, which are treated as fitting parameters in this work.

The volume-dependent phonon frequency of the *i*th mode at ***q*** (i.e., ω_***q***,*i*_) is fitted with the Grüneisen model. The mode-specific Grüneisen parameter γ_***q***,*i*_ is obtained with^59^

4 $$\gamma_{q,i}=-\frac{\ln\left[\omega_{q,i}\left(V\right)\right]-\ln\left[\omega_{q,i}\left(V_{0}\right)\right]}{\ln\left(V\right)-\ln\left(V_{0}\right)}$$

whose integration gives ω_***q***,*i*_(*V*):

5 $$\omega_{q,i}\left(V\right)=\omega_{q,i}\left(V_{0}\right){\left(\frac{V}{V_{0}}\right)}^{-\gamma_{q,i}}$$

where *V*_0_ is the athermal equilibrium volume.

In practice, a constant γ_***q***,*i*_ is derived from linear regression of eq 4 at the sampled volume points. To ensure the continuity of phonon modes, modes at step *V* + Δ*V* are sorted with reference to modes at step *V* by maximizing the sum of dot products between their eigenvectors, which is an example of a more general linear-assignment problem:^60,61^

6 $$\max\overset{3N}{\underset{i}{\sum }}\overset{3N}{\underset{j}{\sum }}X_{ij}e_{q}\left(V\right)\cdot e_{q}\left(V+\Delta V\right)$$

where ***e***_***q***_ denotes the phonon eigenvectors of the ***q***-phased dynamical matrix. **X**_*ij*_ is the Boolean matrix whose element *x*_*ij*_ = 1 when the *j*th mode at *V* + Δ*V* is assigned to the *i*th mode at *V*.

Upon establishing the volume dependence of *U*_DFT_ and ω, the Gibbs free energy *G* at the given temperature *T* and pressure *p* can be minimized to get the equilibrium volume *V*_0_:

$$G\left(T,p\right)=\underset{V}{\min}\left[U_{\mathrm{DFT}}\left(V\right)+F_{\mathrm{vib}}\left(V,T\right)+pV\right]$$ 7

### Equilibrium Lattice

For the proposed QHA LD workflow, volumetric thermal expansion plays a fundamental role, since all thermodynamic properties are expressed as functions of *V*. However, due to the relatively low lattice symmetry, thermal expansions of a wide range of molecular crystals, including monoclinic FI and orthorhombic FII studied here, are highly anisotropic, which requires lattice matrix **L** for more-accurate predictions. Various methods have been adopted in the literature to express and minimize *G* with respect to **L**, including fitting strain-dependent phonon frequencies,^62,63^ numerically integrating thermal gradients,^59^ or minimizing the second-order Taylor-expanded free-energy PES.^64^ These fully anisotropic methods inevitably necessitates an exhaustive configuration search in the high-dimensional space, whose dimensionality increases as the symmetry of the system reduces, escalating the number of phonon calculations required for a stable fitting.

To avoid high computational costs, an extra fitting step is appended to the volume-based workflow, which utilizes lattice parameters of the volume points sampled for fitting ω­(*V*). Anisotropy is accounted for at these points, since their geometries, including lattice and atomic coordinates, are fully relaxed subject to the constant-volume constraint. The symmetrically nonequivalent elements of sampled lattice matrices are independently fitted as polynomials of *V*, then the equilibrium lattice **L**_0_ can be obtained once *V*_0_ is derived from eq 7. *V* is also adopted as a constraint to reduce the number of unknowns and to ensure that the determinant of **L** is equal to *V*. For the monoclinic FI:

8 $$L\left(V\right)=\left(\begin{array}{lll} a\left(V\right) & 0 & 0\\ 0 & b\left(V\right) & 0\\ c_{x}\left(V\right) & 0 & V/ab \end{array}\right)$$

and for the orthorhombic FII:

9 $$L\left(V\right)=\left(\begin{array}{lll} a\left(V\right) & 0 & 0\\ 0 & b\left(V\right) & 0\\ 0 & 0 & V/ab \end{array}\right)$$

### Computational Details

crystal23 ^65–67^ is adopted for calculations based on the linear combination of atomic orbitals (LCAO) method, while quantum espresso 7.3.1 ^68,69^ is adopted for plane-wave calculations based on the projected augmented wave (PAW) method. The standard PAW pseudopotentials parametrized with the PBE functional are employed, which are available in PSlibrary.^70^ Details of SCF and geometry optimization setups are available in Section S1 in the electronic Supporting Information (ESI).

QHA calculations are performed with the “thermodynamics” module of crystalpytools,^61^ based on a set of six equally spaced volume points, with variations ranging from −4% (contracted) to 6% (expanded) of the equilibrium volume. At each volume point, harmonic phonon frequencies are calculated with finite displacements in the central difference formalism, with space group symmetry fully exploited to minimize the number of displacements. Dynamical matrices based on LCAO–DFT are obtained with crystal,^71^ while those based on PAW-DFT are obtained with phonopy.^72,73^ To compensate for the systematically overestimated vibrational frequencies caused by HA and the residual errors on DFT energy surfaces, harmonic frequencies of intramolecular modes are uniformly scaled by factors listed in Table S1 in the ESI.

Dielectric responses and Raman intensities based on LCAO–DFT are computed analytically with the coupled-perturbed Hartree–Fock/Kohn–Sham (CPHF/KS) method implemented in crystal,^74–76^ while a numerical far-from-resonance approximation implemented in phonopy–spectropy^77^ is adopted for PAW-DFT, where the static dielectric tensors of displaced structures are calculated by the density functional perturbation theory (DFPT) implemented in quantum espresso. The parameters adopted to compute the vibrational and spectral properties are documented in Section S1 in the ESI.

Representative CPU times for the main computational steps are summarized in Section S1.6 in the ESI, with additional timing analysis reported in our previous work.^45^

## Results

FI and FII both contain hydrogen-bonded layers (NH···OH and OH···O=C) of molecules, which stack along the crystallographic direction *b* through London dispersions (Figure 1ab). The equilibrium phase under ambient conditions, FI, is characterized by corrugated hydrogen bond networks, which increase interlayer friction and thus cause difficulties in milling and tableting.^2,3^ In contrast, the hydrogen bond networks of the metastable phase, FII, adopt a nearly flat configuration, making this phase more desirable for manufacturing. Previous experimental research has documented the stability of FI over FII for all temperatures up to the melting point.^78^ The stability of FI may be attributable to its larger entropy originating from the higher vibrational freedom of intermolecular phonon modes.^79^ It is apparent that to capture the delicate polymorphic stability of molecular crystals at finite temperatures, thermal effects on their structural, vibrational, and thermochemical properties must be carefully computed. This necessitates the careful documentation of the influence of critical computational parameters, including basis set, Hamiltonian, and the underpinning implementation of QHA LD on the precision of these predictions. Considering the previously established convergence from atomic basis sets to the nearly complete plane-wave basis set,^45^ results based on def2-TZVP and PAW are presented here. Details of results with alternative approximations are presented in the ESI. This ensures the conciseness of the main text and better clarifies the intermingled sources of errors.

**Figure 1:**
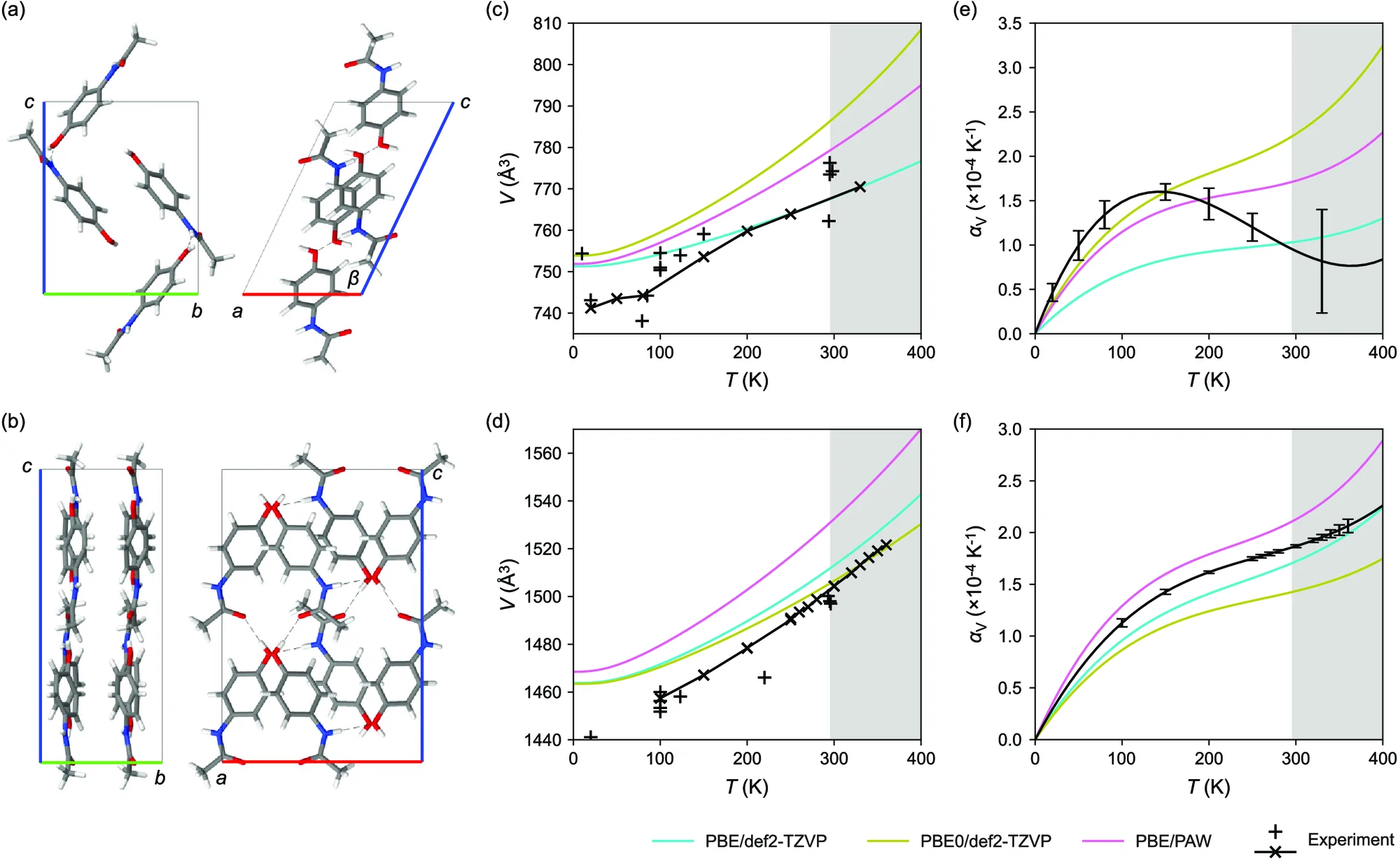
Unit cells of (a) the monoclinic form I and (b) the orthorhombic form II paracetamol polymorphs. White, gray, blue, and red colors respectively denote hydrogen, carbon, nitrogen and oxygen atoms. Hydrogen bonds are denoted by the black dashed lines. Temperature dependence of the equilibrium volume *V* and the volumetric thermal expansion coefficient α_*V*_ of (c, e) form I and (d, f) form II paracetamol. For experimental references, the uncertainty of fitting is annotated with error bars (Section S2.2 in the ESI). The gray-shaded areas are beyond the empirical validity limit of quasi-harmonic lattice dynamics.

### Structural Properties

Volumetric expansion is taken as the starting point as it acts as the fundamental material descriptor of the proposed QHA LD framework. The predicted evolutions of equilibrium volume *V* and volumetric thermal expansion coefficient α_*V*_ with temperature *T* ranging from 0 to 400 K are shown in Figure 1c–f. Individual measurements from various sources are annotated with the plus sign (“+”),^80–99^ whereas two consecutive temperature-dependent measurements of FI^100^ and FII^101^ are plotted as black continuous lines with the cross sign (“×”). All the adopted experimental data are documented in detail in Section S2.1 in the ESI. The consecutive measurements are fitted as fourth-order polynomials to offer references for α_*V*_ (Section S2.2 in the ESI).

The predicted *T*–*V* curves below 200 K are generally similar to each other, while significant differences are noticed within the shaded areas beyond 300 K. This is expected since this is empirically the temperature at which anharmonic contributions intensify and therefore the validity limit of QHA LD (∼^2^/_3_ *T*_m_, *T*_*m*_ ≈ 443 K).^102,103^ The closest agreement with the references is observed for PBE/def2-TZVP. However, the predicted equilibrium volumes deviate more prominently from the references at lower temperatures and, consequently, the gradients of *T*–*V* curves based on PBE/def2-TZVP are lower than the measured trends. In comparison, slightly larger deviations of no more than 2% are obtained with PBE/PAW for both FI and FII, which are relatively uniformly distributed throughout the temperature range, leading to better predictions of the expansion rate. Including Fock exchange exerts limited influence on volume predictions of FII, as suggested by the *T*–*V* curve obtained with PBE0/def2-TZVP. In contrast, with the same set of parameters, disproportionally large thermal expansions are observed for FI at higher temperatures.

To better discriminate errors associated with athermal equilibrium geometry from those due to lattice vibrations, *T*–α_*V*_ curves are illustrated in Figure 1e, f. Notable discrepancies among *T*–α_*V*_ curves for FI are reported for various parametrizations, which become increasingly significant beyond 200 K. In comparison, the predictions for FII are overall consistent and differences only intensify beyond the validity limit of QHA LD. Results based on PBE/PAW best agree with the references thanks to the uniformly distributed errors in *V* throughout the temperature range, indicating that the thermal effects at finite temperatures are well-captured with PBE/PAW. PBE/def2-TZVP underestimates the α_*V*_ of both FI and FII, which corresponds to the lower gradients of *T*–*V* curves. Nevertheless, in both cases, the experimentally observed larger α_*V*_ of FII than that of FI at 300 K^101^ is reproduced. The failure of PBE0/def2-TZVP should be attributed to the thermal activation of fictitiously softened phonon modes in FI, suggesting potential errors in fitted frequencies, which will be discussed in detail in the following section. In addition, the large decrease in reference α_*V*_ of FI between 150 and 300 K is only partially characterized by the reduced gradients in the predictions. Currently, the available measurements are probably insufficient to facilitate detailed analysis,^100^ as indicated by the large error bars in Figure 1e, and a more detailed explanation is available in Section S3.1 in the ESI.

Compared to their volumetric counterparts, the linear expansions of FI and FII exhibit larger deviations from the references (Figure S6), which is not surprising, considering the limited number of sampling points. In particular, the distribution of errors are noticed to be anisotropic. The most prominent difference from the reference lies in *b* of both FI and FII, while for FII, the observed contraction along *c* is reproduced by none of the adopted parametrizations. For comparison, a fully anisotropic QHA method yields limited improvements (Figure S7), probably indicating inherent errors underlying the theoretical framework. While errors along *b* originate from the London dispersion between hydrogen bond networks, which are stacked perpendicular to *b* of both FI and FII, the origin of thermal contraction along *c* of FII is more delicate. A previous study based on X-ray diffraction attributed this contraction to increased distortions in the skeleton of paracetamol molecule at high temperatures, which synergy with the packing motif of FII and reduce the length of the *c*-axis.^101^ Both phenomena are strongly correlated with the configuration of constituent molecules (i.e., atomic coordinates), which are vulnerable to thermally activated intermolecular modes with relatively low frequencies.^34^ This necessitates anharmonic free energy methods that explicitly include variable atomic coordinates. In contrast, thermal effects on atomic coordinates can only be indirectly accounted for by the varied chemical environment within the lattices sampled and optimized at different volumes, which is insufficient according to the predicted linear expansions. A dedicated discussion is attached in Section S3.3 in the ESI, revealing the prevalence and potential influences of this drawback. For CSP tasks with softer and more flexible molecules, such effects can probably become even more prominent.^34^

Ideally, atomic coordinates can be explicitly taken into account via free-energy minimization on higher-dimensional PES, where iterative calculations of structure and phonons are adopted, but this requires exceptional computational resources that are usually beyond the capacity of modern high-performance computers for crystals with more than one hundred atoms.^28,104^ As an essential compromise for practicability, an extra constant-volume optimization step is performed based on the equilibrium volume predicted by QHA LD and the athermal PES, whose results are compared to both the fitted and experimental data to examine the potential improvements. The predicted finite-temperature lattice constants of FI and FII are listed in Table 1.

**Table 1:** Finite-Temperature Geometries of Form I and Form II Paracetamol^a^

Polymorph	Parameterization	Volume (Å^3^)	***a*** (Å)	***b*** (Å)	***c*** (Å)	**β** (deg)
form I	PBE/def2-TZVP	763.92 (0.00)	7.07 (−0.18, 0.07)	9.21 (−1.09,-0.20)	12.92 (0.65, 0.10)	114.83 (−0.67, −0.02)
form I	PBE0/def2-TZVP	778.79 (1.95)	7.14 (0.79,-0.01)	9.42 (1.10,-0.21)	12.90 (0.53, 0.11)	116.16 (0.49, −0.10)
form I	PBE/PAW	773.33 (1.23)	7.05 (−0.55, 0.02)	9.35 (0.39, 0.02)	12.92 (0.71, 0.10)	114.76 (−0.72, 0.15)
form I	Experiment^b^	763.9	7.086(2)	9.315(2)	12.833(3)	115.60(2)
						
form II	PBE/def2-TZVP	1510.85 (0.43)	11.67 (−1.39, 1.51)	7.48 (0.95,-1.82)	17.31 (0.88, 0.20)	
form II	PBE0/def2-TZVP	1506.39 (0.13)	11.76 (−0.68, 1.90)	7.44 (0.52,-2.03)	17.21 (0.29, 0.17)	
form II	PBE/PAW	1532.43 (1.86)	11.66 (−1.48, 1.21)	7.58 (2.39,-1.21)	17.33 (0.97, 0.01)	
form II	Experiment^c^	1504.4(7)	11.837(7)	7.4057(13)	17.162(3)	

a Percentage errors against experimental and QHA data (ESI) are reported respectively as the first and second values in parentheses, when applicable. Otherwise the error against experimental data is reported. Geometries at 250 K­(300 K), 0 GPa are reported for the form I (II) paracetamol.

b HXACAN18 ^100^ (Table S3). The standard uncertainty of the last digit is reported in parentheses.

c HXACAN23 ^101^ (Table S4). The standard uncertainty of the last digit is reported in parentheses.

Deviations from experiments are generally comparable among all parametrizations presented, since basis set completeness has been established previously as the dominant factor underlying geometry predictions, and the def2-TZVP basis set can obtain results similar to a plane-wave description.^45^ Despite the overestimated expansion of FI by PBE0/def2-TZVP, results under the temperature studied (250 K) are not severely deteriorated. Another interesting observation is the negligible differences between optimized lattice parameters of FI and their counterparts based on QHA LD, which probably indicates the equivalence of both methods when the variation in molecular configurations is limited. The differences exerted by constrained optimization are more notable for FII due to the higher degrees of freedom in molecular configurations, and the optimization partially corrects the predictions of lattice vectors *a* and *b*. However, this effect is marginal and not generally applicable to all parametrizations and lattice constants (Tables S6 and S7 in the ESI), so it can be concluded that the constrained optimization at predicted volume exerts negligible influences on the agreement between QHA LD and experiments. On the other hand, the consistent results obtained by QHA LD and the extra optimization step also indicate that they are fundamentally based on the same athermal PES.

To summarize, the plane-wave basis set achieves optimal accuracy for structural property predictions, where the size of basis set becomes crucial. The def2-TZVP basis set can obtain comparable results in ideal cases, but its results are vulnerable to errors in volume-dependent phonon frequencies, as indicated by the case of FI based on PBE0/def2-TZVP. In addition, the variable molecular configuration is highlighted as an important source of error undermining the prediction of lattice constants. This reveals an inherent limitation that challenges the accuracy of current QHA LD workflow, and further theoretical developments are probably required.

### Vibrational Properties

Lattice vibrations play a fundamental role in QHA-derived properties, providing insight into both the microscopic interactions and the macroscopic properties of crystalline materials. Results in the previous section have revealed that errors in phonons can severely deteriorate the estimated structural properties, since the induced noise in free energy inevitably alters the fundamental temperature–volume–pressure relationship of QHA thermodynamics. Therefore, robust fitting of volume-dependent phonon frequency is the prerequisite for reliable property predictions. As an important factor underpinning the quality of mode-specific Grüneisen fittings, in Section S3.4 in the ESI, the continuity of phonons throughout the sampled volume range is discussed based on the coefficient of determination *R* ^2^. The choice of basis set primarily determines the distribution of *R* ^2^, where the plane-wave basis set clearly outperforms all the atomic basis sets tested. This observation stresses the necessity of using large, diffuse basis sets to minimize noises on free energy PES, whereas even the def2-TZVP basis set is proven insufficient, despite the comparable structural property predictions to those based on plane-wave.

Ideally, a robust QHA fitting is expected to reproduce exactly the harmonic phonon modes calculated at the corresponding volume, considering the identical PES underlying both methods. This consistency can be utilized to obtain a wider range of properties at the QHA level, such as Raman spectra, which require phonon eigenvectors that are absent within the current QHA LD framework. In Figure 2a,b, the QHA-derived phonon frequencies ν_QHA_ are compared to their counterparts ν_HA_ based on harmonic LD and the corresponding lattice optimized at 300 K. The presented results are limited within the scope of low-frequency intermolecular vibrations (i.e., 0–250 cm^–1^), whose thermal activation is the key contributor to solid-state transitions, including thermal expansion and polymorphic phase transition.

**Figure 2:**
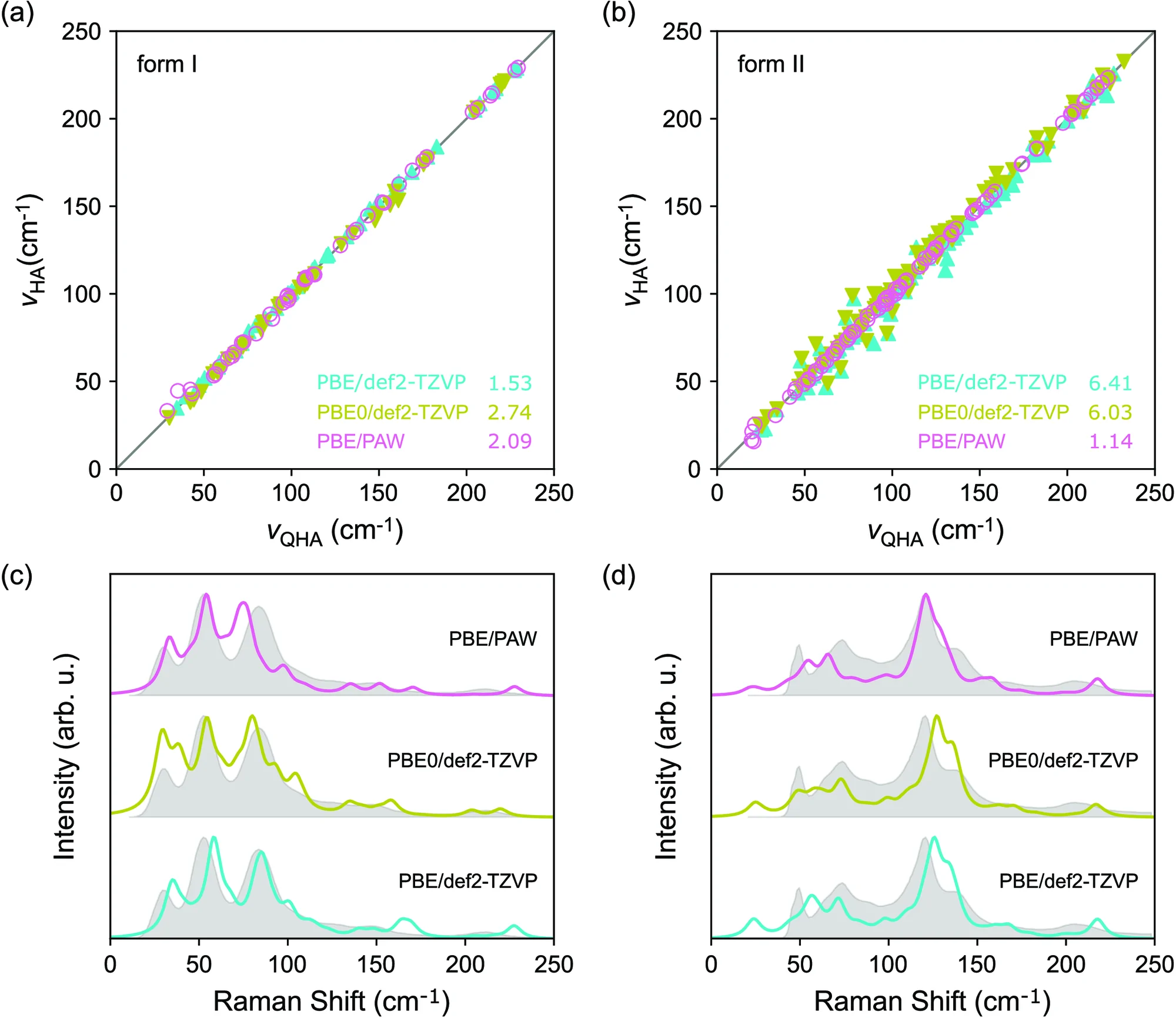
Harmonic intermolecular phonon frequencies ν_HA_ at the equilibrium volume of 300 K for (a) form I and (b) form II paracetamol, as functions of quasi-harmonic phonon frequencies ν_QHA_ obtained at the same volume. Root-mean-square-deviations in cm^–1^ are annotated with legends. Experimental and calculated Raman spectra of (c) form I and (d) form II paracetamol between 0 and 250 cm^–1^ at 300 K. For comparison, the experimental references are replicated as gray-shaded areas in the background, and the calculated intensities are normalized to the highest peak of each plot. [Experimental spectra adapted with permission from ref 105. Copyright 2012, American Chemical Society.]

With atomic basis sets, ν_QHA_ and ν_HA_ of FI show close proximity, while the distribution becomes more scattered for FII, leading to larger root-mean-square-deviations (RMSD). This probably originates from the higher flexibility in the molecular configuration of FII (Section S3.3 in the ESI), which increases the Pulay force that QHA LD fails to account for and consequently generates errors in free-energy PES. In contrast, the plane-wave basis set achieves optimal alignment between ν_QHA_ and ν_HA_, since its basis functions are defined with respect to the lattice cell. Though the cell also changes during constant-volume optimization, the case of paracetamol, as a relatively rigid molecule, suggests that the Pulay forces induced by variable atomic coordinates are the dominant source of error in calculating quasi-harmonic phonons for crystals consisting of more flexible molecules.

Beyond the agreement among theoretical models, Figure 2c, d presents the simulated polycrystalline Raman spectra between 0 and 250 cm^–1^ of FI and FII, whose agreement with experimental measurements^105^ are evaluated. With thermal expansion considered, the predicted Raman spectra are generally red-shifted because of the reduced intermolecular bindings that soften vibrations. Therefore, agreement with experiments is remarkably improved compared to those based on harmonic LD and athermal equilibrium geometries,^45^ suggesting the effectiveness of the current QHA LD workflow for practical CSP tasks to predict the vibrational properties of molecular crystals under ambient conditions. Raman spectra based on PBE/PAW and PBE/def2-TZVP exhibit a high degree of mutual consistency, despite the relatively poor phonon continuity and the more scattered ν_QHA_–ν_HA_ plot of the latter. This probably indicates that the phonon calculation at QHA-derived volume yields a subtle correction effect for atomic basis sets, increasing the result alignment with plane-wave. By comparing the spectra predicted by PBE/def2-TZVP and PBE0/def2-TZVP, it is clear that influences of Fock exchange are more prominent for the intensities rather than the positions of Raman peaks, highlighting the potential cruciality of Hamiltonian for (hyper)­polarizability, whose predictability, however, is beyond the current scope.

Results in this section stress the substantial impact of basis set completeness in ensuring phonon continuity throughout the volume range, which can reduce the noises on free-energy PES and ensure a numerically stable QHA fitting. From this prospective, the plane-wave basis set yields the optimal performance, achieving a good convergence between the fitted (ν_QHA_) and the calculated (ν_HA_) phonon frequencies, which are respectively based on the analytical QHA LD and the numerical harmonic LD. Nevertheless, results obtained by def2-TZVP are improved via optimization and phonon calculation at QHA-derived volumes, leading to comparable Raman spectra to those based on plane-wave. This opens the potential for further refinement of spectra predictions with more-sophisticated functionals, considering the non-negligible differences in Raman intensities upon inclusion of Fock exchange.^106^

### Thermochemical Properties

The general-purpose and reliable polymorph free-energy ranking workflow remains a key challenge in CSP, as energy prediction is usually influenced by intertwined errors in geometry, lattice energy, and phonons. Moreover, the weak noncovalent interactions within molecular crystals yield subtle energy differences, necessitating a quantitatively accurate method that can provide sufficient resolution. To assess the performance of QHA LD based on different basis sets and Hamiltonians, a number of measurable thermochemical properties are calculated under standard conditions, including the constant-pressure specific heat *C*_*p*_^°^, and the Gibbs free energy Δ*G*_sub_^°^, enthalpy Δ*H*_sub_^°^, and entropy Δ*S*_sub_^°^ of sublimation. The calculated data are compared with published measurements^107–110^ in Table 2.

**Table 2:** Thermochemical Properties of Form I and Form II Paracetamol at 298.15 K, 101.3 kPa

Polymorph	Parameterization	*C*_p_^°^ (J mol^–1^K^–1^)	Δ*G*_sub_^°^ (kJ mol^–1^)	Δ*H*_sub_^°^ (kJ mol^–1^)	Δ*S*_sub_^°^ (J mol^–1^K^–1^)
form I	PBE/def2-TZVP	179.55	66.92	135.14	228.81
form I	PBE0/def2-TZVP	188.99	57.22	126.05	230.88
form I	PBE/PAW	183.80	60.50	130.13	233.52
form I	Experiment	163 ± 1^a^	60.0^a^	117.9 ± 0.7^a^	190 ± 2^a^
		189.68^b^		129.9 ± 1.4^c^	
form I	Theory^d^		58.8–80.7	131.0–154.1	242.1–246.3
					
form II	PBE/def2-TZVP	186.04	67.95	134.16	222.08
form II	PBE0/def2-TZVP	184.43	58.07	125.55	226.35
form II	PBE/PAW	189.33	62.41	129.39	224.65
form II	Experiment	191.5^b^	56.1^b^	115.9 ± 0.9^a^	200 ± 3^a^

a See refs 107 and 108.

b See ref 109.

c See ref 110.

d See ref 28. In total, five different sets of parametrization were tested using plane-wave density functional and fragment-based post Hartree–Fock methods. Ranges of reported data are presented here. Δ*G*_sub_^°^ is obtained using Δ*G*_sub_^°^ = Δ*H*_sub_^°^ – 298.15Δ*S*_sub_^°^.

In light of the relatively large discrepancy of 27 J mol^–1^ K^–1^ in the measured *C*_p_^°^, data reported by Boldyreva et al.^109^ can probably be regarded as upper boundaries. Then, from this perspective, *C*_p_^°^ by PBE/def2-TZVP and PBE/PAW agrees reasonably well with the reference, given the differences of no more than 10 J mol^–1^ K^–1^ below the boundary. In contrast, despite the fact that PBE0/def2-TZVP also obtains similar values respectively for FI and FII, it fails to reproduce the relative difference between two polymorphs. This probably originates from the numerical instabilities previously identified for FI based on PBE0/def2-TZVP, as *C*_p_^°^ is the second derivative of Gibbs free energy and might be vulnerable to noise on free-energy PES.

The inclusion of Fock exchange remarkably improves the predicted Δ*G*_sub_^°^, as evidenced by the decreases of over 9 kJ mol^–1^ from PBE/def2-TZVP to PBE0/def2-TZVP, leading to better alignments with reference for both FI and FII. Literature results on other hydrogen-bonded molecular clusters suggest the delicate differences between DFT and hybrid Hamiltonians in calculating the binding energies, which should be attributed to the inherent self-interaction error of semilocal DFT Hamiltonian when describing electron densities within hydrogen bonds.^10,40^ In addition, increasing the size of the basis set also yields marginal corrections to the overestimated Δ*G*_sub_^°^, as indicated by results based on PBE/PAW. However, this should probably be attributed to the moderately expanded volumes (Table 1) that reduce intermolecular bindings, rather than more-accurate descriptions of electron or phonon structures, as suggested by the following results of Δ*H*_sub_^°^ and Δ*S*_sub_^°^.

Δ*H*_sub_^°^ directly includes static internal energy, where more prominent influences of the Hamiltonian tend to manifest. In contrast, Δ*S*_sub_^°^ is purely based on phonon frequencies, where, as aforementioned, the basis set is expected to be the dominant factor. Despite relatively large deviations from experiments, the predicted Δ*H*_sub_^°^ and Δ*S*_sub_^°^ agree reasonably well with theoretical references, probably indicating similar underlying sources of error. As commented by McKinley and Beran,^28^ the measured Δ*H*_sub_^°^ might be insufficient to offer solid references given the difference of ∼12 kJ mol^–1^ between different sources, whereas theoretical predictions of Δ*H*_sub_^°^ and Δ*S*_sub_^°^ are also found vulnerable to errors in thermal expansions. Therefore, the following analysis focuses on the consistency among the theoretical data. The predicted Δ*H*_sub_^°^ seem to be simultaneously affected by the choices of basis set and Hamiltonian, where results based on PBE/def2-TZVP differ from those obtained with PBE/PAW and PBE0/def2-TZVP by approximately 5 kJ mol^–1^ and 9 kJ mol^–1^, respectively. Nevertheless, all the relative differences in Δ*H*_sub_^°^ between FI and FII are around 0.5–1.0 kJ mol^–1^, suggesting a uniform distribution of errors in FI and FII. Close proximity is observed among all Δ*S*_sub_^°^, where negligible differences of no more than 5 J mol^–1^ K^–1^ lead to limited scatterings within 1.5 kJ mol^–1^ for the entropic term under standard conditions. This probably originates from error cancellations between the entropies of crystalline and molecular phases. Furthermore, all theoretical data suggest the higher Δ*S*_sub_^°^ for FI, reversing the experimentally observed relative stability.^79,107^ This error is believed to originate from the insufficiently described quasi-free rotations of methyl group within molecular crystals,^111^ which counterbalances the correct relative difference in Δ*H*_sub_^°^ and propagates to Δ*G*_sub_^°^, fictitiously stabilizing FII under standard conditions. Further discussion of this error is attached in Section S3.7 in the ESI.

As the quantity directly associated with polymorph energy rankings, the Gibbs free energy is prominently influenced by the Hamiltonian, where the inclusion of Fock exchange improves the agreement with references. However, other thermochemical functions studied here are generally under the impacts of complex and intermingled sources of errors, impeding accurate property prediction in practical CSP tasks. A controllable and relatively uniform distribution of these errors probably requires both large basis set and hybrid Hamiltonian, necessitating a composite workflow to balance the computational costs.

## Discussion

The above analysis sheds light on the dominant sources of errors that impede accurate property prediction for molecular crystals. The importance of basis set choice has been highlighted in almost all properties studied, where the plane-wave basis set achieves the optimal performance. In comparison, the macroscopic structural and spectral properties based on the def2-TZVP basis set largely equate to those by plane-wave, but the cross-volume continuity and fitting quality of phonon modes in the microscopic scale are deteriorated due to its limited size. The influence of the Hamiltonian manifests in computing thermochemical properties such as Gibbs free energy, since Fock exchange can help to reduce the electron self-interaction error of gradient-corrected density functionals. It is clear that, for molecular crystals, the quality of basis set dominates the accuracy of phonon-related properties, whereas the inclusion of Fock exchange improves the description of electron-related properties. This raises the question whether the efficiency of QHA LD can be enhanced by rationally combining computational parameters according to the property of interest. Several composite schemes have been proposed for QHA LD in the literature, which usually implement various levels of theory for geometry, static internal energy, and phonons to reduce overall time consumption.^57,112^ However, this inevitably alters the underlying PES during fittings, whose inconsistent descriptions can induce numerical noise and consequently lead to errors. In ref 15, a composite QHA LD workflow consisting of PBE-based phonon and PBE0-based static internal energy failed to predict the relative stability of α-, β-, and γ-glycine, while both PBE0-based QHA LD and composite HA LD workflows obtain qualitatively correct results.

Based on the results presented here, a promising approach is the consistent adoption of a combination of plane-wave basis set and the gradient-corrected density functional, which ensures efficiency while minimizing noise. For properties where the influences of Hamiltonian are non-negligible, such as energies,^28,40,41^ (hyper)­polarizability^106^ or magnetism,^113–115^ constrained optimization and harmonic phonon calculation using hybrid functional at QHA-derived volumes is then required for greater accuracy. Considering the infeasible cost of plane-wave hybrid functional calculations, this is only practical via atomic basis sets that obtain comparable geometry and harmonic phonons, where, as established here and in our previous work,^45^ the def2-TZVP basis set is a pertinent candidate. Figure 3 provides a graphical description of the optimal workflow proposed.

**Figure 3:**
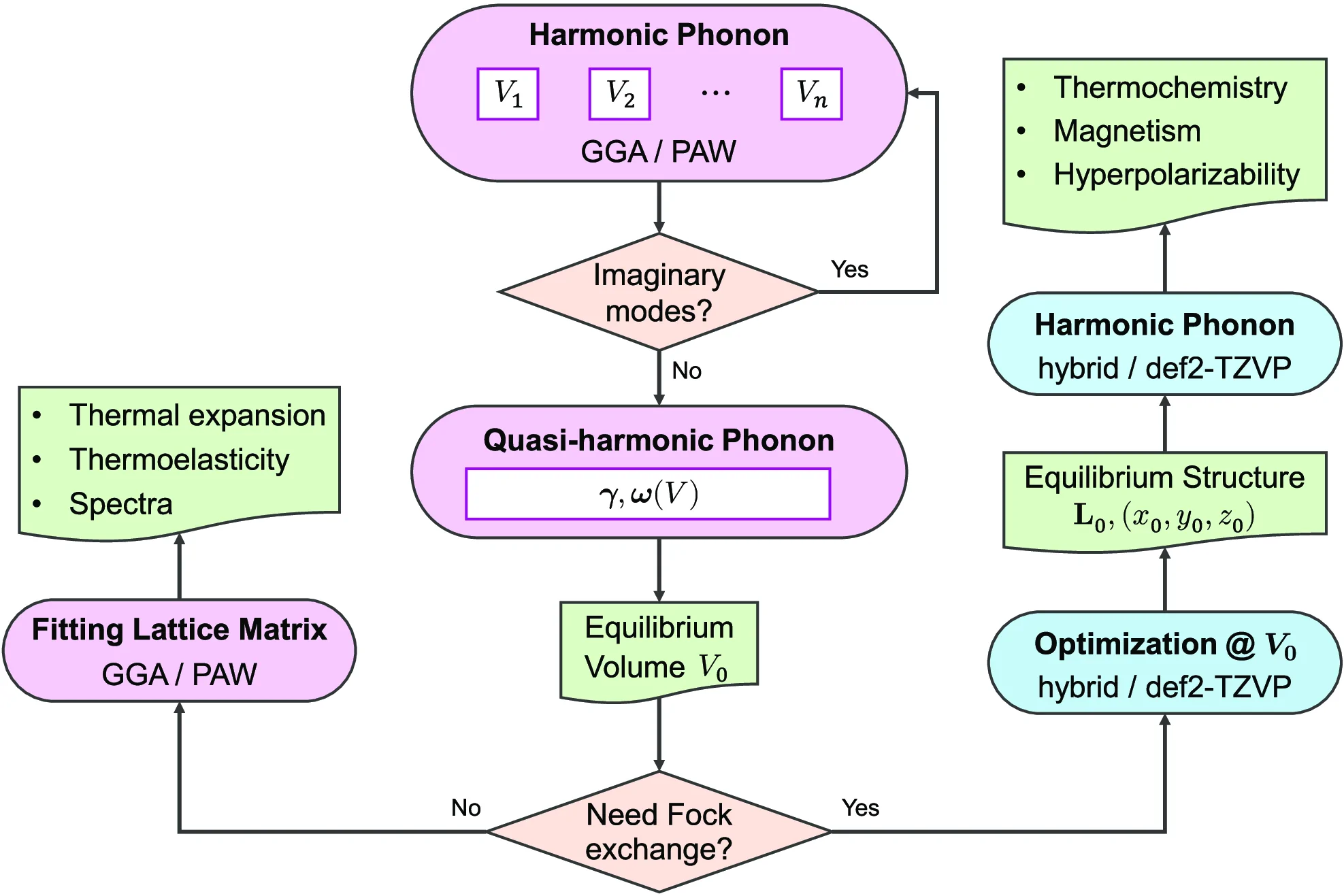
A composite workflow for quasi-harmonic lattice dynamics.

For validation, the proposed composite workflow is implemented utilizing the equilibrium volumes derived from QHA LD and PBE/PAW at 300 K, based on which harmonic phonons are computed with PBE0/def2-TZVP. The intermolecular Raman spectra of FI and FII, respectively obtained with PBE/PAW, PBE0/def2-TZVP and the composite workflow, are visualized in Figure 4a,b. Peak positions predicted by the composite workflow show close proximity to those based on PBE/PAW and the references, suggesting that harmonic phonons of the workflow benefit from the improved geometry predictions of the plane-wave basis set. The inclusion of Fock exchange improves agreement with experimental spectra around 80 cm^–1^ for FI and 140 cm^–1^ for FII, where the corresponding phonon modes exhibit strong coupling.^64^ As revealed previously,^45^ spectra based on PBE and PBE0 are shifted differently by such anharmonicity, and this study further suggests that the latter yields better outcomes. Therefore, the proposed workflow can benefit from the synergy of large atomic basis set and hybrid Hamiltonian while keeping the computational cost affordable, significantly improving the accuracy of vibrational property predictions.

**Figure 4:**
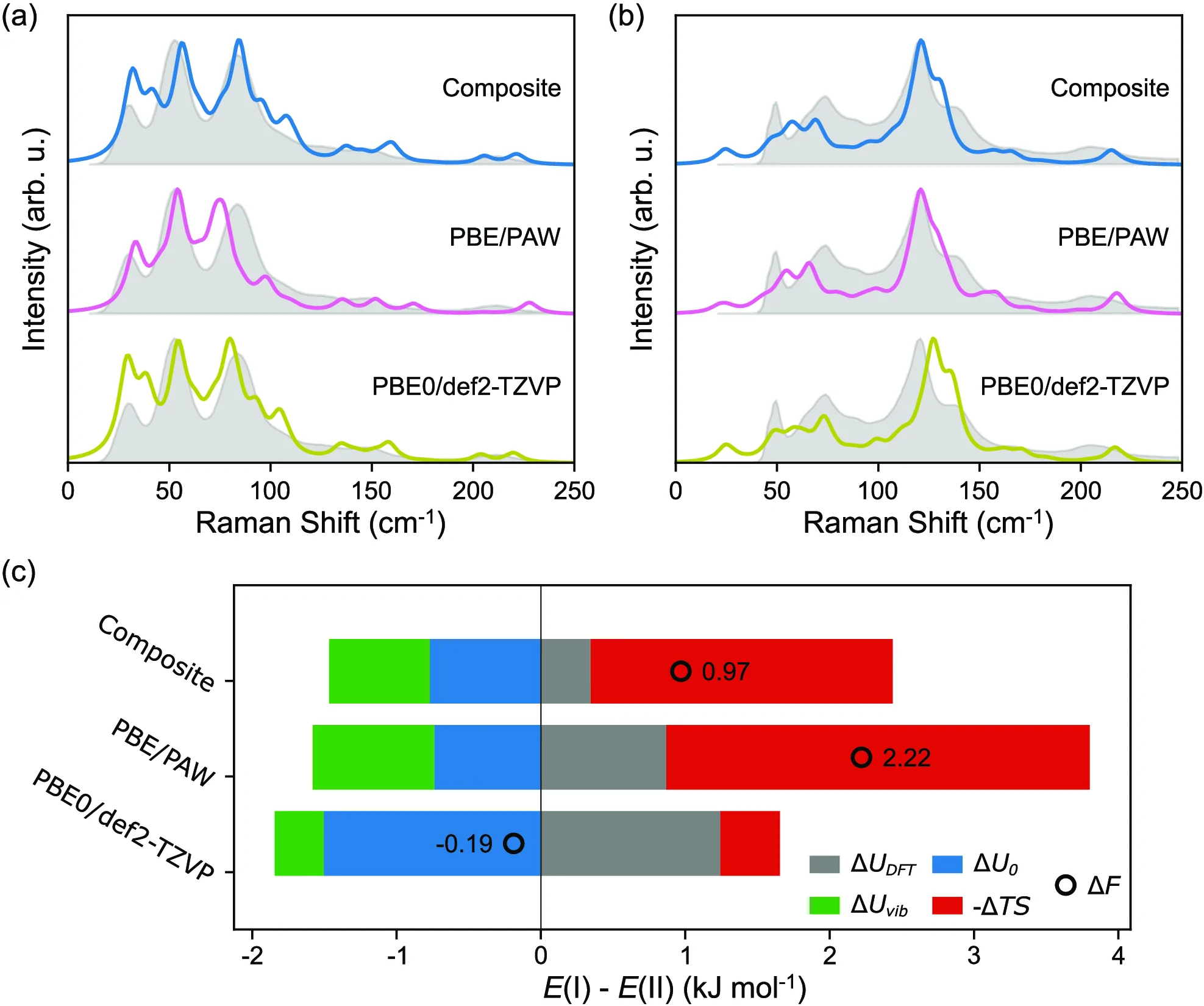
Raman spectra of (a) form I and (b) form II paracetamol between 0 and 250 cm^–1^ at 300 K. All the annotations are consistent with those in Figure 2c,d. [Experimental spectra adapted with permission from ref 105. Copyright 2012, American Chemical Society.] (c) Differences in Helmholtz free energy (Δ*F*) between form I and form II paracetamol at 300 K. Δ*F* is partitioned respectively into the differences of static internal energy Δ*U*_DFT_, zero-point energy Δ*U*_0_, thermal vibrational energy Δ*U*_vib_, and entropic term −Δ*TS*. All the values are reported in kJ mol^–1^ per formula unit.

Both Δ*G*_sub_^°^ and Δ*H*_sub_^°^ predicted by the workflow (Table S13 in the ESI) agree well with experimental references, which achieve values similar to those based on the much more time-consuming PBE0/def2-TZVP while maintaining the accuracy of other properties at the PBE/PAW level. In contrast, the workflow makes negligible differences in Δ*S*_sub_^°^. The failure to align theoretical Δ*S*_sub_^°^ with measurements, as well as the failure to reproduce the Δ*S*_sub_^°^ difference between FI and FII, is primarily associated with the aforementioned methyl group dynamics. As analyzed in Section S3.7 in the ESI, this indicates a central limitation of the present QHA LD based on Cartesian displacement sampling, and can only be addressed by techniques beyond conventional QHA LD, such as curvilinear coordinate sampling or MD-based free-energy methods. Since this error is inherent for QHA LD, probably very limited, if any, improvement can be made within the theoretical framework discussed in this study.

Since polymorph free-energy ranking is the practical target of the proposed workflow, the difference in Helmholtz free energy between FI and FII Δ*F* at 300 K is further compared in Figure 4c for the workflow and its parent methods. Δ*F* is partitioned into differences in static internal energy Δ*U*_DFT_, zero-point energy Δ*U*_0_, thermal vibrational energy Δ*U*_vib_, and entropic term −Δ*TS*, allowing the dominant source of error in the free-energy ranking of FI and FII to be identified. Compared with PBE/PAW, the proposed workflow shifts the predicted Δ*F* toward the experimental ordering, demonstrating that the improved structural, vibrational, and thermochemical descriptions propagate into the polymorph energy ranking. Nevertheless, the workflow still fails to correctly reproduce the relative stability of FI and FII. The remaining discrepancy is largely associated with the entropic term that artificially stabilizes FII over FI and dominates Δ*F*, and the workflow is insufficient to reverse this trend. In contrast, PBE0/def2-TZVP gives the correct qualitative ordering, but Figure 4c shows that this agreement arises from cancellation among differently distributed errors, including the underestimated −Δ*TS*, overestimated Δ*U*_0_, and the overly expanded FI volume discussed above. According to Figure S14 in the ESI, excessive entropic stabilization of FII is a general trend for all DFT parametrizations tested, which originates from the lack of anharmonic and configurational effects (e.g., methyl group rotation and temperature-dependent molecular configuration) within the current QHA LD framework.

Compared to PBE/PAW, the proposed workflow shifts the predicted free-energy difference toward experimental ordering, although it still fails to correctly reproduce the relative stability of FI and FII. The residual discrepancy is largely associated with the entropic term that excessively stabilizes FII due to the neglected anharmonicity and configurational effects, including methyl group rotation and temperature-dependent molecular configuration. The free-energy differences between pharmaceutical polymorphs are typically on the order of only 1–2 kJ mol^–1^, where anharmonicity of a comparable, or even greater, magnitude may arise. According to a previous study on the relatively rigid succinonitrile polymorphs, anharmonicity shifts the free-energy difference by 1.55 kJ mol^–1^.^34^ This emphasizes that (i) the numerical precision of PES is not necessarily the dominant source of error in practical polymorph ranking, and (ii) the overall accuracy can be hindered by how the PES is sampled, i.e., the specific free-energy method adopted. Therefore, the proposed workflow can be adopted to define the converged limit for a chosen combination of basis set and Hamiltonian, rather than as a complete treatment of all finite-temperature contributions to polymorph stability. With the precision of underlying PES firmly established, standard MD-based free-energy methods such as thermodynamic integration can provide reliable complementary routes to address the remaining anharmonicity.

## Conclusions

In this work, QHA LD is adopted with a hierarchy of parametrizations to study the structural, vibrational, and thermochemical properties of paracetamol polymorphs at finite temperatures and pressures. Our results systematically present the best affordable numerical precision of QHA LD for polymorphic molecular crystals. Based on these findings, a composite QHA LD workflow involving at most two sets of parametrization is proposed, which promotes predictive material modeling for a wide range of applications while ensuring efficiency.

The present work identifies the error induced by the neglected configurational freedom of constituent molecules as an inherent drawback of QHA LD. Other simulation techniques, such as MD, are probably required to improve the geometry predictions of molecular crystals. The failure to reproduce the entropy difference between FI and FII is also highlighted, as it is a direct source of the reversed polymorph energy ranking. This necessitates the detailed energy profile of free-rotating groups such as methyl to fully account for the rotational entropy associated. The current sampling scheme of conventional QHA LD based on Cartesian displacements is insufficient to fully account for this effect, where MD can be a suitable alternative. From this perspective, this work offers valuable references for rational parametrization of ab initio MD. It also facilitates the efficient generation of high-quality training datasets for machine-learning force fields, considering the numerical precision achieved here.

The key findings of present work are expected to be transferable to other polymorphic molecular crystals, but this should be interpreted in light of the benchmark system adopted here. Paracetamol is a useful prototype for crystalline pharmaceutical compounds, because of the presence of representative interactions including London dispersion, π–π stacking, hydrogen bonds and methyl group dynamics, while being experimentally well-characterized. Nevertheless, it remains a relatively rigid molecule compared with much larger target compounds encountered in recent CSP blind tests,^33,116^ where substantial conformational flexibility, orientational disorder, and prominent anharmonic motions may lead to a different hierarchy of errors and complicate polymorph ranking. Therefore, the present results should not be taken to imply that basis set and Hamiltonian effects dominate the predictions of all properties for all molecular crystals, which is inaccurate even for the relative stability between FI and FII, as implied in Figure 4c. Instead, the present work identifies how specific DFT parametrizations influence QHA LD predictions. In future studies on more flexible, disordered or anharmonic systems, the proposed workflow, which efficiently controls the precision of DFT-derived PES, provides a solid reference for more-advanced free-energy methods.

In conclusion, this work offers a detailed analysis of basis set and Hamiltonian effects on DFT modeling of polymorphic molecular crystals. An optimal QHA LD workflow is proposed to balance the cost and performance of practical CSP tasks within quasi-harmonic framework, facilitating future research on molecular crystals with higher complexity. The limitations of QHA LD identified here can inspire the proposal and development of correction schemes, offering a practical parametrization strategy for subsequent anharmonic or MD-based free-energy calculations.

## Supplementary Material





## Notes

Further details on computational setups; tabulated experimental data referenced by this work; additional results and discussions (PDF)
Dumped HA/QHA LD outputs in YAML format, generated and read by crystalpytools (ZIP)
