# Supplementary material for: Predictive Modeling of Paracetamol Polymorphs: Linking Basis Set and Hamiltonian Effects to an Optimized Quasi-Harmonic Lattice Dynamics Workflow
Source: J Chem Theory Comput. 2026 Sep 9;22(18):9626–39. doi: 10.1021/acs.jctc.6c00750 (PMC13602900; doi:10.1021/acs.jctc.6c00750)
Supplement: Supplementary file 1 [file ct6c00750_si_001.pdf]

Supporting Information: *Predictive modeling of paracetamol polymorphs: Linking basis set and Hamiltonian effects to an optimized quasi-harmonic lattice dynamics workflow*

Huanyu Zhou,*,†,‡ Giuseppe Mallia,† and Nicholas M. Harrison†

†*Department of Chemistry, Imperial College London, White City Campus, 80 Wood Lane, London, W12 0BZ, United Kingdom*

‡*School of Aerospace Engineering and State Key Laboratory of Environment Characteristics and Effects for Near-space, Beijing Institute of Technology, Beijing, 100081, China*

E-mail: huanyu.zhou20@imperial.ac.uk

Contents

1 Computational details **S3**

1.1 Pseudopotentials . . . S3

1.2 Self-consistent field (SCF) . . . S3

1.3 Geometry optimization . . . S5

1.4 Harmonic and quasi-harmonic lattice dynamics . . . S6

1.5 Raman spectra . . . S8

1.6 Approximate CPU time . . . S9

2 Statistical treatment of experimental data **S10**

2.1 Lattice parameters . . . S10

2.2 Thermal expansions . . . S13

2.3 Elastic bulk moduli . . . S14

3 Supplementary results **S15**

3.1 Bulk expansions and isothermal moduli . . . S15

3.2 Linear expansions and lattice constants . . . S19

3.3 Variations in molecular configuration . . . S25

3.4 Phonon continuity . . . S27

3.5 Intermolecular vibrations . . . S29

3.6 Intramolecular vibrations . . . S32

3.7 Thermochemical properties . . . S35

3.8 The composite workflow . . . S40

S1 Computational details

S1.1 Pseudopotentials

CRYSTAL23 (C23) is used for density functional theory (DFT) calculations based on the all-electron linear combination of atomic orbitals (LCAO) method, and Quantum Espresso 7.3.1 (QE) is used for DFT calculations based on the projected augmented wave (PAW) method. PAW pseudopotentials parameterized with the generalized gradient approximated Perdew-Burke-Ernzerhof (GGA-PBE) functional and published as PSlibrary are adopted for PAW-DFT, including:[1]

- Oxygen: 'O.pbe-n-kjpaw_psl.1.0.0.UPF'
- Nitrogen: 'N.pbe-n-kjpaw_psl.1.0.0.UPF'
- Carbon: 'C.pbe-n-kjpaw_psl.1.0.0.UPF'
- Hyrogen: 'H.pbe-kjpaw_psl.1.0.0.UPF'

The results reported in the manuscript suggest negligible differences between all-electron and pseudopotential DFT calculations in predicting structural and vibrational properties of form I (FI) and form II (FII) paracetamol crystals. Considering the dominating non-covalent interactions within the lattices of FI and FII, the more significant role played by valence electrons in both London dispersions and hydrogen bonds might explain this phenomenon.

S1.2 Self-consistent field (SCF)

In both LCAO-DFT and PAW-DFT calculations, a 4×4×2 Monkhorst-Park grid is used to sample the first Brillouin zone of FI and FII. The variation in total energy per cell during SCF and geometry optimization is converged to 1×10^{-9} Hartree (*i.e.*, 2×10^{-9} Ry for PAW-DFT).

The SCF input block for C23 is specified below:

```
DFTD3
VERSION
4
ABC
END
GCPAUTO
TOLDEE
9
FMIXING
50
TOLINTEG
8 8 8 8 24
SHRINK
0 4
4 4 2
ENDSCF
```

Standard parameterizations of the geometrical counterpoise (gCP) correction are adopted unaltered for all atomic basis sets in this work. As presented in the example above, this is achieved with the shorthand keyword `GCPAUTO` for calculations based on def2-SVP and def2-TZVP basis sets, while the following inputs set gCP corrections for 6-31G** and pob-TZVP basis sets, respectively:

```
GCP
METHOD
DFT/631GD
END
```

```
GCP
METHOD
DFT/POBTZ
END
```

For the most diffusive def2-TZVP basis set, the following keywords are adopted to reduce the numerical instabilities arising from the pseudo-linear dependence problem.

```
LDREMO
10
TOLINTEG
8 8 8 8 30
```

For QE, the cutoff energies of electron wave function and charge density are set to 100 Ry and 400 Ry respectively, achieving an accuracy of at least 3.3×10^{-6} Ry per atom. The following input blocks are used:

```
&SYSTEM
  ecutwfc          = 100
  ecutrho          = 400
  occupations      = 'fixed'
  input_dft        = 'pbe'
  vdw_corr         = 'dft-d3'
  dftd3_version    = 4
  dftd3_threebody  = .TRUE.
/
&ELECTRONS
  conv_thr         = 2.0D-9
/
ATOMIC_SPECIES
  O  16.00  O.pbe-n-kjpaw_psl.1.0.0.UPF
  N  14.01  N.pbe-n-kjpaw_psl.1.0.0.UPF
  C  12.01  C.pbe-n-kjpaw_psl.1.0.0.UPF
  H   1.008 H.pbe-kjpaw_psl.1.0.0.UPF
K_POINTS automatic
  4 4 2 0 0 0
```

S1.3 Geometry optimization

The equilibrium lattice parameters and atomic coordinates of FI and FII are fully relaxed while keeping the space group symmetry. All geometry optimizations are performed with the Broyden-Fletcher-Goldfarb-Shanno (BFGS) method until the energy gradients are less than 1×10^{-4} Hartree Bohr^{-1}.

The following inputs are used for the `OPTGEOM` keyword of C23:

```
OPTGEOM
TOLDEG
0.00010
TOLDEX
0.00040
ENDOPT
```

For FII based on PBE/PAW, `forc_conv_thr` is reduced to 0.5×10^{-4} Hartree Bohr^{-1} (*i.e.*, 1×10^{-4} Ry Bohr^{-1}) to eliminate imaginary frequencies of expanded structures.

The following inputs are used for QE:

```
&CONTROL
  calculation    = 'vc-relax'
  etot_conv_thr = 1.0D-5
  forc_conv_thr = 1.0D-4
  tprnfor        = .true.
  tstress        = .true.
```

```
/
&IONS
  ion_dynamics = 'bfgs'
  upscale      = 100
/
&CELL
  cell_dynamics  = 'bfgs'
  cell_dofree    = 'ibrav'
/
```

The expanded and contracted geometries are optimized with volume constraints. For C23, the `CVOLOPT` keyword is inserted to the `OPTGEOM` input block. For QE, `cell_dofree = 'ibrav'` is replaced by `cell_dofree = 'ibrav+shape'`.

S1.4 Harmonic and quasi-harmonic lattice dynamics

The finite displacement method with the '2-step' central difference formalism is adopted for harmonic lattice dynamics. The resultant Hessian matrices are symmetrized according to the space group symmetry.

For LCAO-DFT calculations, this is achieved with the `FREQCALC` keyword of C23:

```
FREQCALC
NUMDERIV
2
ENDFREQ
```

For PAW-DFT calculations, PHONOPY is used. The displacement step length of FII is increased to 0.06 Bohr to reduce noises on the potential energy surface (PES). Such inconsistent parameterization casts marginal influences on phonon vibrations, as has been analyzed in detail in the **Section S1.3** of our previous work.[2] Besides, the authors noticed that a new Hessian matrix symmetrization scheme derived from the projector-based method as implemented in SYMFC[3] has been made default since PHONONPY 2.30, which is not available at the preparation stage of this manuscript. Strictly symmetrizing the Hessian matrix according to permutation symmetry rules, sum rules and symmetry group operators might reduce noises on PES when smaller step lengths are used, whereas in this work, Hessian matrices are symmetrized with the systematic displacement method. The following

configuration is used for displacement generation by `phonoy-load`:

```
DIM                     = 1 1 1
CREATE_DISPLACEMENTS    = .TRUE.
DISPLACEMENT_DISTANCE = 0.06
```

The following configuration is used for diagonalization of Hessian matrices:

```
FORCE_CONSTANTS = WRITE
MESH            = 1 1 1
GAMMA_CENTER    = .TRUE.
EIGENVECTORS    = .TRUE.
```

To correct the inherent errors of the harmonic PES and to offset the systematic blue shifts of the Raman spectra, the same set of uniform frequency scaling factors as those used in our previous work[2] is adopted throughout this work to correct intramolecular vibrations beyond 250 cm^{-1}, which is summarized in **Table S1**.

Table S1: Frequency scaling factors

	**6-31G****	**def2-SVP**	**pob-TZVP**	**def2-TZVP**	**PAW**
**PBE0**	0.9509	0.9555	0.9594	0.9591	
**PBE**	0.9848	0.9904	0.9923	0.9923	1.0

Given the presence of a free-rotating methyl group, the quasi-rigid rotor harmonic oscillator (quasi-RRHO) approach proposed by Grimme[4] is adopted during the vibrational analysis of gaseous molecules. The entropy of paracetamol molecule is corrected with this approach, so the sublimation entropy of FI and FII are indirectly influenced. The quasi-RRHO approach is implemented using an in-house developed script, which is available on request.

The general procedures for lattice dynamics (LD) based on the quasi-harmonic approximation (QHA) have been introduced in detail in the manuscript, where six volume points are sampled at -4%, -2%, 0%, 2%, 4% and 6% of deformation around the fully optimized athermal equilibrium volume. For the pob-TZVP basis set, significant numerical instabilities are observed in the PES of form II paracetamol with 6% expansion, probably due to its reduced diffuseness of valence orbitals compared to other molecular counterparts, such

as def2-TZVP, as analyzed in our previous work.[2] Therefore, QHA calculations with the pob-TZVP basis set are fitted based on five volume points of deformations ranging from -4% to +4%. To examine its convergence, the number of volume points is further reduced to four, which include harmonic phonons sampled at -2%, 0%, 2% and 4% of deformations. In **Table S2**, the properties of FII at 300 K, 0 GPa are taken as the test case, where results of 5-point fitting are compared with those based on 4-point fitting. Differences in volume ΔV and static internal energy ΔU_{DFT}, as well as root-mean-square-deviation (RMSD) in phonon frequencies are reported.

Table S2: Convergence test of volume points

**Parameterization**	ΔV **(%)**	ΔU_{DFT} **(kJ mol^{-1})**	**RMSD (cm^{-1})**
PBE0/pob-TZVP	0.00	0.03	1.01 (1.36, 0.66, 1.63)[a]
PBE/pob-TZVP	-0.07	-0.22	1.58 (0.98, 1.44, 2.48)[a]

[a] Root-mean-square-deviation (RMSD) throughout the frequency range. RMSDs respectively in the low-, medium-, and high-frequency regions (defined in **Section S3.5**) are reported in the parentheses.

It is clear that for both PBE0/pob-TZVP and PBE/pob-TZVP, the removal of one volume point for fitting does not severely deteriorate the predictions of the structural, vibrational, and thermochemical properties of FII. Errors induced by fewer sampling points are negligible compared to those originating from the pob-TZVP basis set itself, which are elucidated in result analysis.

S1.5 Raman spectra

For dielectric tensors, default convergence thresholds are adopted for (hyper)polarizabilities of C23 calculations based on the coupled-perturbed Hartree-Fock/Kohn-Sham (CPHF/KS) method. The convergence threshold is tightened to 10^{-15} for the linear response potential of QE calculations with density functional perturbation theory (DFPT).

For comparisons with experimental Raman spectra, the calculated intensity of a Raman active mode m is scaled by a factor depending on the temperature of measurement T and

the angular frequency of the incoming laser ω_0:

$$I_m = \frac{(\omega_0 - \omega_m)^4}{c^4} \frac{\hbar}{\omega_m} \left[\frac{1}{\exp\left(\frac{\hbar\omega_m}{k_B T}\right) - 1} + 1 \right] I'_m \tag{S1}$$

where I_m is the experimental Raman intensity of mode m, and I'_m is its theoretical counterpart obtained from the polarizability tensor $\boldsymbol{\alpha}$ and mode eigenvector $\boldsymbol{e_m}$, *i.e.*, $I'_m \propto (\boldsymbol{\alpha}/\partial \boldsymbol{e_m})^2$. ω_m is the angular frequency of mode m. $\hbar$, c and k_B denote the reduced Planck constant, the vacuum speed of light, and the Boltzmann constant, respectively. In this work, the experimental conditions by Nanobolu and Burley are adopted, where $T = 300$ K, $\omega_0 = 2\pi c/\lambda = 3540.70$ THz.[5]

S1.6 Approximate CPU time

Considering the size of target systems, directly comparing the total time consumption of QHA LD calculations is not straightforward, because these calculations typically consist of multiple independent harmonic phonon calculations, with the requested number of nodes and walltime adjusted according to resource availability and level of theory. Nevertheless, in Table S5 of our previous work,[2] the CPU time per SCF step t_{CPU} and the number of SCF steps n_{SCF} preceding each phonon calculation were used as an approximate cost metric. This offers important references to estimate the cost of present study, since the same target system, parameterization, and computing service were used by both studies. All calculations were performed on the ARCHER2 UK National Supercomputing Service (https://www.archer2.ac.uk) with 128 cores (2×AMD EPYC 7742 64-core 2.25GHz processors) per node.[6]

The adopted central-difference finite-displacement scheme requires 120 'energy+gradient' calculations for both FI and FII with their space group symmetry fully utilized. Therefore, $t_{CPU} \times n_{SCF} \times 120 \times 6$ provides a rough estimate of the total cost of a QHA LD workflow sampled at six volume points, neglecting variations caused by volume or symmetry.

The cost of proposed composite workflow can also be assessed with previously reported t_{CPU} and n_{SCF}. The computational load of PBE0/def2-TZVP is approximately 48 times that of PBE/PAW for FI and 6 times for FII. The different use of symmetry in Quantum Espresso and CRYSTAL may lead to disproportional costs for FI and FII. The proposed workflow consists of six PBE/PAW volume points and one PBE0/def2-TZVP refinement point, while a comparable PBE0/def2-TZVP workflow requires six volume points and one refinement point. Therefore, it is estimated that the proposed workflow requires only $(6 + 48)/(48 \times 7) \approx 0.16$ times of the cost of a PBE0/def2-TZVP workflow for FI and $(6 + 6)/(6 \times 7) \approx 0.29$ for FII. Compared to the PBE/PAW workflow, the proposed workflow is approximately 7.71 times more expensive for FI and 1.71 times for FII. This additional cost is justified by the improved free energy descriptions from hybrid DFT while retaining the advantages of plane-wave basis set for geometry and phonon.

PBE0/PAW calculations for FI and FII at the same level of precision as the cases presented in the manuscript are restricted by available computational resources. For an isolated paracetamol molecule, the total CPU time of a converged SCF calculation is 68.87 s for PBE0/def2-TZVP using 128 cores on ARCHER2. In comparison, 985.22 s is required for PBE0/PAW based on identical computational setups and a 20Å×20Å×20Å cubic cell.

S2 Statistical treatment of experimental data

S2.1 Lattice parameters

The experimental lattice parameters and the corresponding temperatures of measurement are collected from the Cambridge Structural Database (CSD),[7] which are summarized in **Table S3** and **Table S4** respectively for FI and FII. Various representations of the periodic cell were adopted in different sources. For consistency, the $P\,1\,2_1/c\,1$ representation of space group 14 is adopted for FI, and the $P\,2_1/b\,2_1/c\,2_1/a$ representation of space group 61 is adopted for FII.[8] FINDSYM is used to standardize space group representations.[9] The

structural data are rounded to three decimal places when available; otherwise the original data are reported.

Table S3: Experimental lattice constants of form I paracetamol

**CSD code**	***T*** **(K)**	***a*** **(Å)**	***b*** **(Å)**	***c*** **(Å)**	***β*** **(deg)**	***V*** **(Å^3)**
HXACAN01	295	7.10	9.40	12.93	115.9	776.272
HXACAN02	295	7.12	9.40	12.88	116.2	773.466
HXACAN04	150	7.094	9.263	12.811	115.61	759.093
HXACAN06	100	7.082	9.250	12.763	115.52	754.512
HXACAN07	123	7.094	9.232	12.764	115.59	753.937
HXACAN27	100	7.092	9.215	12.742	115.59	750.997
HXACAN28	85	7.077	9.173	12.709	115.57	744.2
HXACAN30	100	7.091	9.211	12.741	115.61	750.39
HXACAN34	298	7.102	9.385	12.892	115.70	774.266
HXACAN35	294	7.066	9.337	12.823	115.71	762.22
HXACAN36	20	7.084	9.173	12.680	115.60	743.11
HXACAN48	10	7.125	9.220	12.726	115.53	754.374
HXACAN57	79.15	7.066	9.194	12.554	115.17	738.096
HXACAN13[a]	20	7.073	9.166	12.667	115.51	741.2
HXACAN14[a]	50	7.073	9.173	12.698	115.51	743.5
HXACAN15[a]	80	7.077	9.173	12.709	115.57	744.2
HXACAN16[a]	150	7.079	9.240	12.769	115.54	753.6
HXACAN17[a]	200	7.084	9.278	12.815	115.57	759.8
HXACAN18[a]	250	7.086	9.315	12.833	115.60	763.9
HXACAN19[a]	330	7.085	9.370	12.872	115.62	770.5

[a] The temperature-dependent measurement used for fitting thermal expansion coefficients.[10]

Table S4: Experimental lattice constants of form II paracetamol

CSD code	T (K)	a (Å)	b (Å)	c (Å)	V (Å^3)
HXACAN	295	11.805	7.393	17.164	1497.98
HXACAN08	123	11.777	7.212	17.166	1458.1
HXACAN25	220	11.764	7.286	17.104	1466.05
HXACAN31	100	11.765	7.188	17.168	1451.80
HXACAN32	100	11.776	7.203	17.168	1456.22
HXACAN33	293	11.824	7.397	17.153	1500.19
HXACAN37	20	11.755	7.139	17.171	1441.12
HXACAN38	100	11.768	7.197	17.162	1453.38
HXACAN41	100	11.76	7.232	17.16	1460
HXACAN45	296	11.806	7.399	17.143	1497
HXACAN21[a]	100	11.782	7.199	17.183	1457.4
- [ab]	150	11.793	7.243	17.175	1467.1
- [ab]	150	11.794	7.243	17.175	1467.1
HXACAN22[a]	200	11.806	7.293	17.169	1478.3
- [ab]	200	11.807	7.293	17.170	1478.5
- [ab]	250	11.818	7.347	17.165	1490.2
- [ab]	250	11.821	7.347	17.166	1490.9
- [ab]	260	11.826	7.358	17.163	1493.5
- [ab]	270	11.823	7.370	17.165	1495.6
- [ab]	280	11.830	7.381	17.164	1498.8
- [ab]	300	11.835	7.405	17.162	1504.3
HXACAN23[a]	300	11.837	7.406	17.162	1504.4
- [ab]	320	11.839	7.431	17.163	1509.9
- [ab]	330	11.847	7.443	17.160	1513.2
- [ab]	340	11.853	7.456	17.159	1516.3
- [ab]	350	11.855	7.468	17.158	1519.1
HXACAN24[a]	360	11.853	7.481	17.160	1521.6

[a] The temperature-dependent measurement used for fitting thermal expansion coefficients.[11]
[b] Not available on CSD.

S2.2 Thermal expansions

To obtain the volumetric thermal expansion coefficients α_V, the analytical expressions of volume as the function of temperature $V(T)$ for FI and FII are established by polynomial fittings. For all fittings, the first-order term is set to zero to keep $\alpha_V = 0$ at zero temperature. The optimal degree of polynomials is evaluated by fitting the experimental measurements, as illustrated in **Figure S1**.

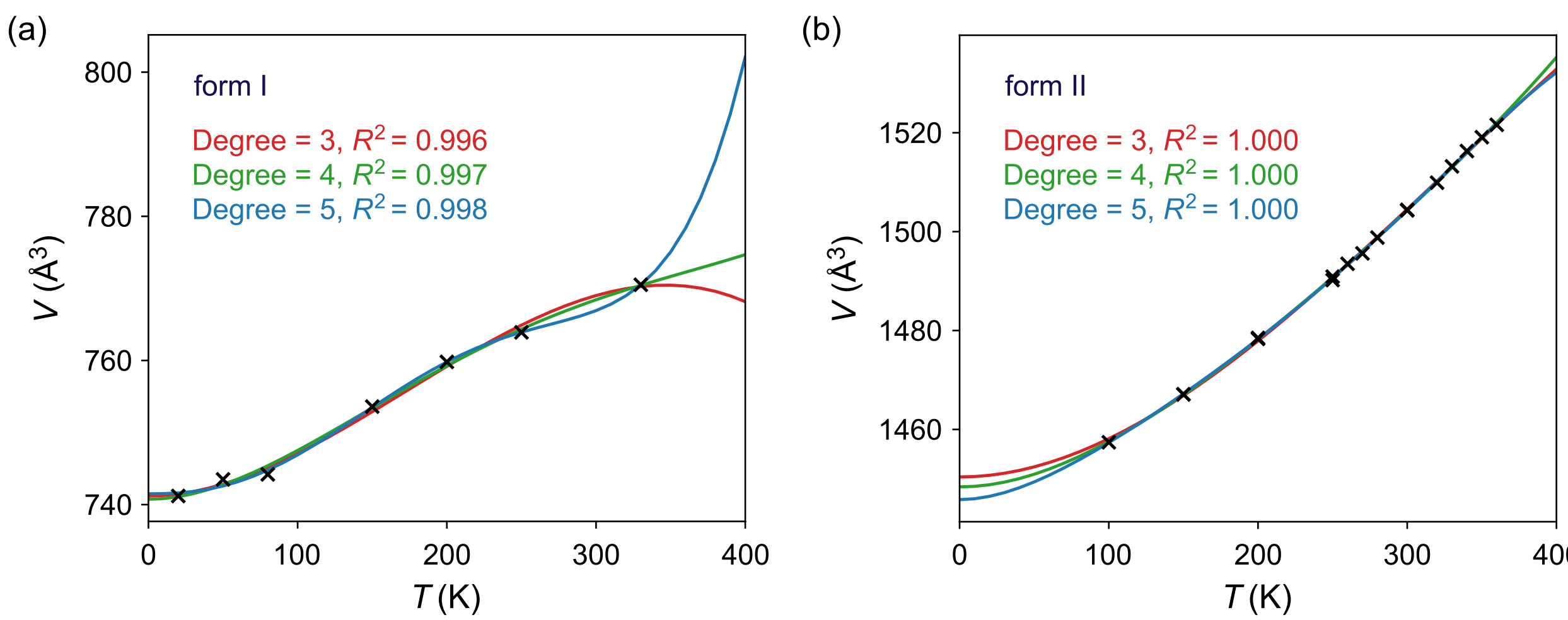

Figure S1: Polynomial fittings of the experimental temperature-volume $(T - V)$ relationship of (a) form I and (b) form II paracetamol. Measurements reported by Wilson (form I)[10] and Drebushchak and Boldyreva (form II)[11] are annotated with black crosses. R^2 is annotated in the legend.

For FI, the fourth-order polynomial exhibits the optimal alignment with experiment, where the low expansion rate within 0 ∼ 100 K and 250 ∼ 330 K is captured. Though a higher R^2 is reported for the fifth-order polynomial, the overfitting error is prominent, which destabilizes the results beyond 330 K. In comparison, all polynomials achieve the nearly identical and idealized $V(T)$ fittings for FII. For consistency, the fourth-order polynomial is adopted in this work. The fitting parameters for experimental data are summarized in **Table S5**, and those fitted for the theoretical data are reported in the YAML dump files as part of the **electronic supporting information (ESI)**.

Errors in the polynomial fittings of $V(T)$ can propagate to $\alpha_V(T)$ curves, as indicated

Table S5: Polynomial fittings of $V(T)$

**Polymorph**	$\boldsymbol{V_0}$ **(Å^3)**	$\boldsymbol{T^2}$ **(Å^3 K^{-2})**	$\boldsymbol{T^3}$ **(Å^3 K^{-3})**	$\boldsymbol{T^4}$ **(Å^3 K^{-4})**
form I	740.7419561	0.00095284	3.0487364×10^{-6}	2.99242614×10^{-9}
form II	1448.3768869	0.00113989	2.4385717×10^{-6}	2.36561312×10^{-9}

by the error bars in **Figure 1** of the manuscript. It should be noted that these error bars indicate the standard error σ induced by fittings, instead of the variability of the original measurements. Under the given temperature T, σ is computed as follows:

$$\sigma(T) = \frac{1}{V(T)}\sqrt{\mathbf{d}\boldsymbol{\Sigma}\mathbf{d}^T} \tag{S2}$$

where $\boldsymbol{\Sigma}$ is the covariance matrix from the polynomial fitting of $V(T)$. $\mathbf{d}$ is a row vector that corresponds to the expression of $V(T)$. Here $\mathbf{d} = (0, 2T, 3T^2, 4T^3)$.

S2.3 Elastic bulk moduli

The experimental bulk moduli B_0 of FI and FII are derived from the isothermal X-ray diffraction measurements of the volume-pressure ($V - p$) curves by Boldyreva *et al.* at 295 K.[12] WebPlotDigitizer was adopted to extract data from figures 1 and 2 of the original publication.[13] The third-order Birch-Murnaghan equation of state (EoS) is adopted for fitting,[14] whose derivative at constant temperature gives:

$$p(V) = \left[\frac{\partial F(V)}{\partial V}\right]_T = \frac{3B_0}{2}\left[\left(\frac{V_0}{V}\right)^{\frac{7}{3}} - \left(\frac{V_0}{V}\right)^{\frac{5}{3}}\right]\left\{1 + \frac{3}{4}\left(B_0' - 4\right)\left[\left(\frac{V_0}{V}\right)^{\frac{2}{3}} - 1\right]\right\} \tag{S3}$$

The extracted data points and fitted curves are illustrated in **Figure S2**. The fitting parameters, including the equilibrium volume V_0, the bulk moduli B_0 (also plotted in **Figure S5**), and its derivative to the pressure B_0' at 295 K and 0 GPa, are annotated with the coefficient of determination R^2.

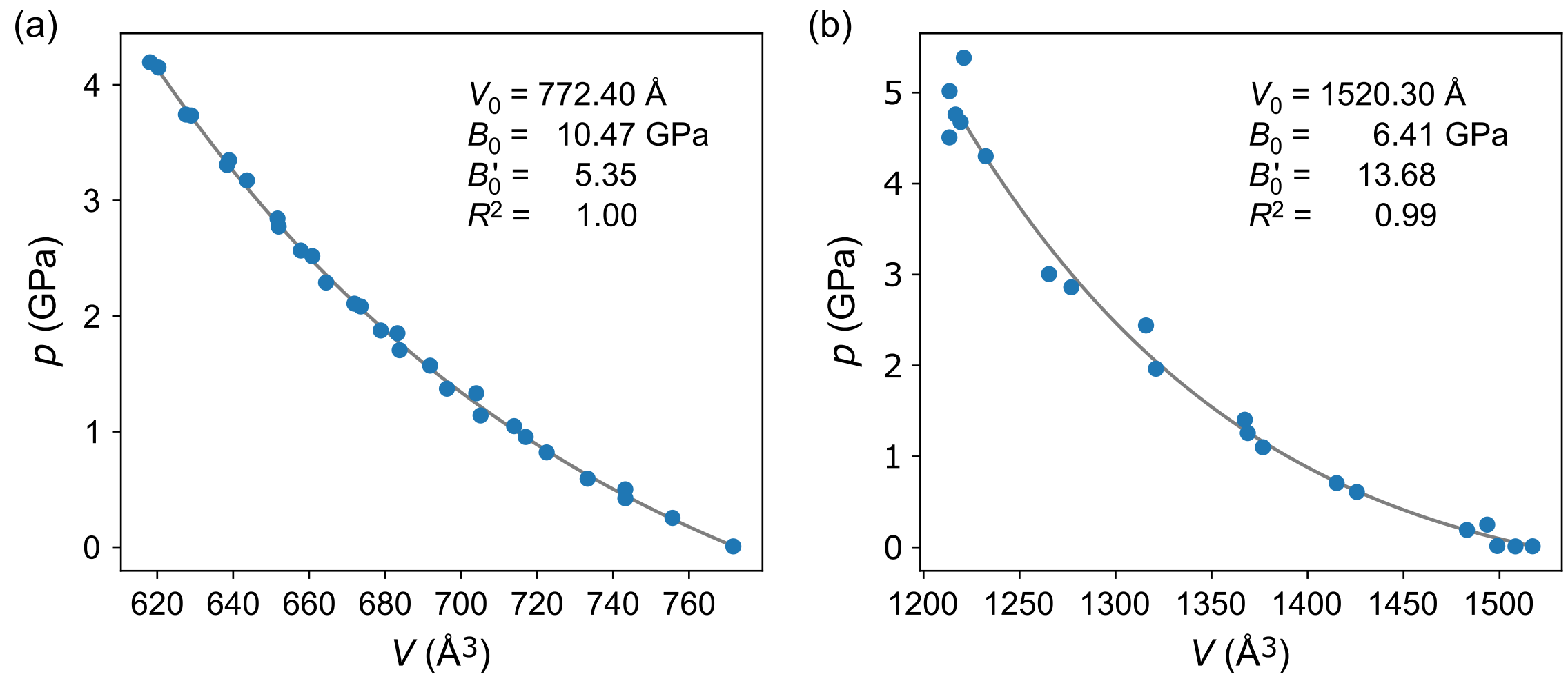

Figure S2: The experimentally measured volume-pressure relationships of (a) form I and (b) form II paracetamol. All measurements were made under ambient conditions. The fitted equations of state are plotted as continuous gray curves, whose parameters are annotated in the upper-right corner of each sub-figure. Experimental data adapted with permission from ref 12. Copyright 2002 Kluwer Academic Publisher/Akadémiai Kiadó.

S3 Supplementary results

S3.1 Bulk expansions and isothermal moduli

The complete set of temperature-dependent equilibrium volumes V and thermal expansion coefficient α_V from 0 K to 400 K of FI and FI are shown in **Figure S3** with experimental references, whose annotations and sources are consistent with those of the manuscript.

V obtained with triple-ζ basis sets, including pob-TZVP and def2-TZVP, are close to the references and those based on the plane-wave basis set. In comparison, double-ζ basis sets significantly overestimate V, where, in particular beyond 300 K, unphysical volumes are predicted for FII when PBE is adopted (**ESI**). This suggests the importance of cardinality for atomic basis sets in predicting volumetric expansions and clearly illustrates that the tested triple-ζ basis sets are comparable in precision to the much larger plane-wave basis set. Similarly to previous observations,[2] the influences of Fock exchange are marginal when the basis set is well-converged. The inclusion of Fock exchange partially corrects the overestimated V of double-ζ basis sets, probably due to the better athermal geometry considering

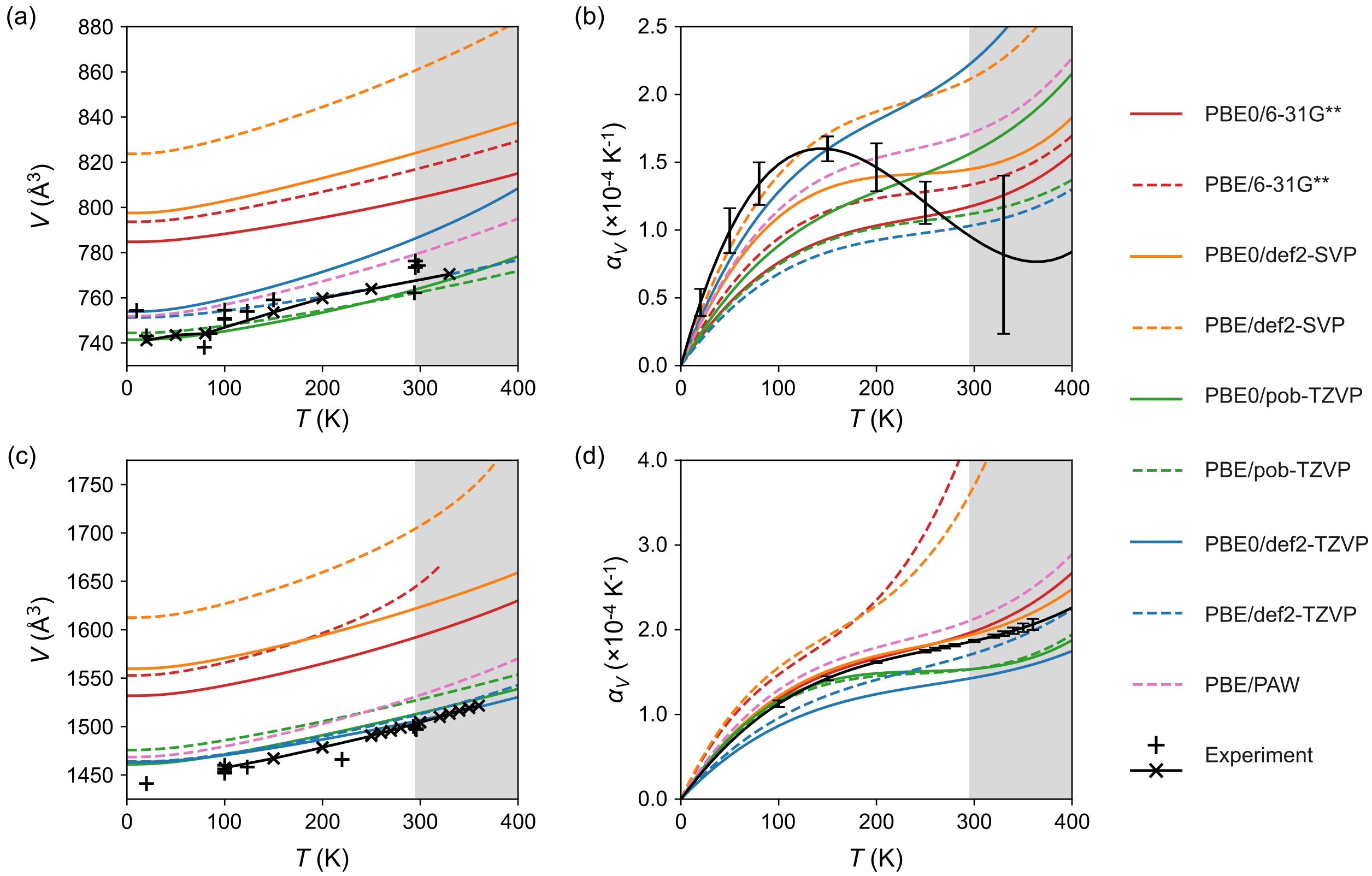

Figure S3: Temperature dependence of the equilibrium volume V and the volumetric thermal expansion coefficient α_V of (a)(b) form I and (c)(d) form II paracetamol. Other marks and annotations are consistent with those in **Figure 1** of the manuscript.

the similar slopes of the $T-V$ curves based on PBE and PBE0. However, its effect remains subordinate, which cannot counterbalance the errors induced by the limited sizes of double-ζ basis sets.

α_V of FI is significantly overestimated by PBE/def2-SVP and PBE0/def2-TZVP especially at higher temperatures, as revealed in **Figure S3b**, while similar behavior is also noticed for FII with PBE/6-31G** and PBE/def2-SVP. The origin of this phenomenon probably lies in the fitting of volume-dependent phonon frequencies as analyzed in the manuscript, which consequently leads to numerical instabilities. In comparison, results based on numerically stable fittings are generally comparable, suggesting athermal geometry as the dominant source of error, especially for double-ζ basis sets.

Regarding the lowered α_V of FI between 150 K and 300 K, it could probably be attributed to the pronounced temperature-dependent anisotropy of non-covalent interactions,

where distinct hydrogen bonds and non-bonded contacts exhibit highly variable expansion rates. However, as pointed out by Wilson,[10] a comprehensive discussion of anisotropic lattice strains is restricted by the limited number of available variable-temperature measurements, making it insufficient to construct a robust parameterization. Besides, polynomial fittings also generate large errors in the reference $T - \alpha_V$ curve, as stated in **Section S2.2**, which means that the amplitude of such decrease might be exaggerated. To avoid the influence of fitting errors during result analysis, the percentage volumetric expansions against low-temperature reference ($\Delta V/V_0$) are presented in **Figure S4**.

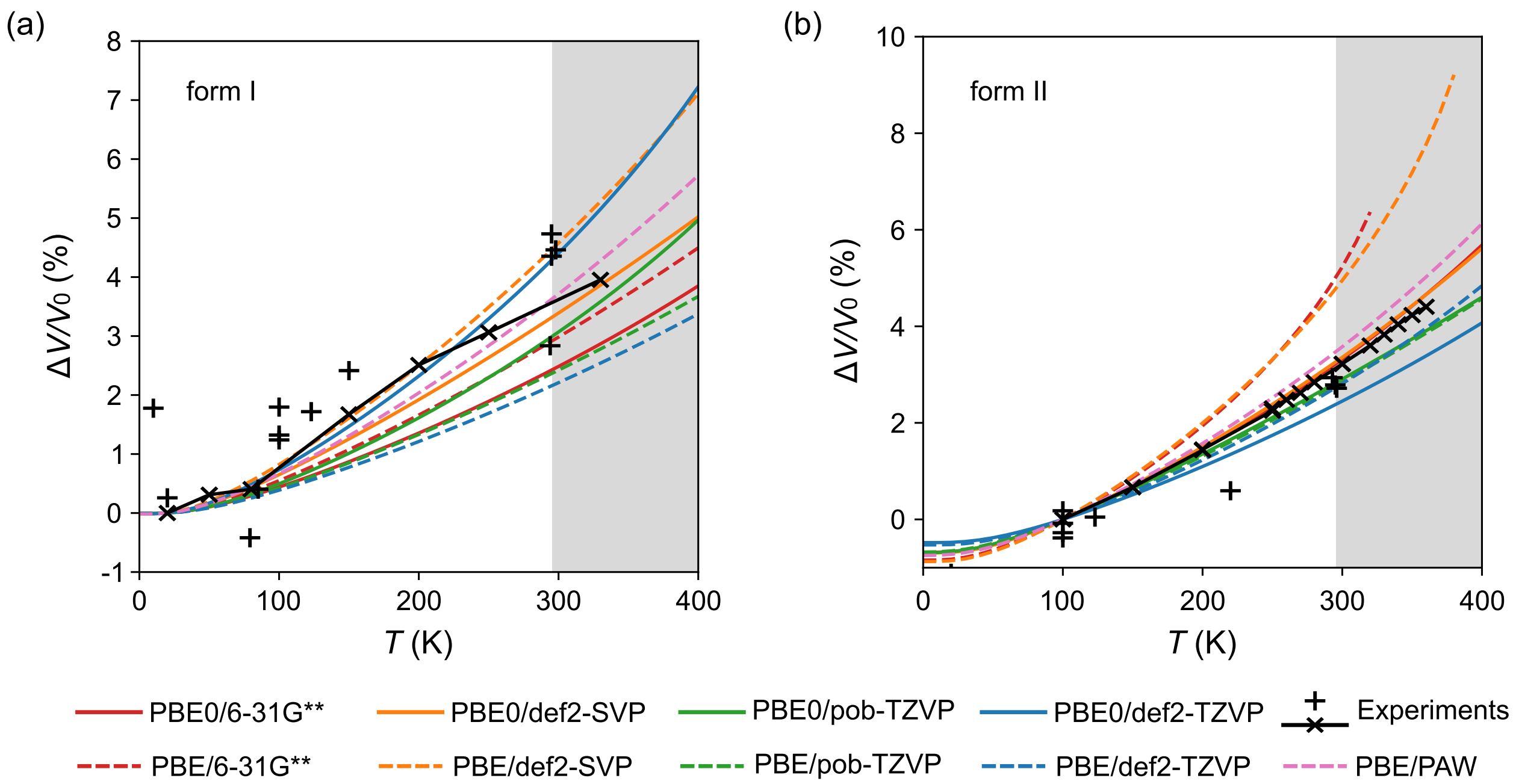

Figure S4: Volumetric expansion (ΔV) as the percentage of low-temperature equilibrium volume (V_0) of (a) form I and (b) form II paracetamol. Due to the limit of available references, V_0 is defined as the equilibrium volume of form I (II) crystal at 20 K (100 K). The gray-shaded areas are beyond the empirical validity limit of quasi-harmonic lattice dynamics.

For numerically stable fittings, the predicted $T - \Delta V/V_0$ curves lie within the range of available references, suggesting that the general treads of thermal expansions of FI and FII have been captured. The better alignment between experimental and theoretical results in **Figure S4** probably indicates that the large decrease in α_V of FI might be an artifact of polynomial fitting. PBE/def2-SVP and PBE0/def2-TZVP are outliers of FI, whereas PBE/6-

31G** and PBE/def2-SVP are outliers of FII. This further supports the analysis in the manuscript that errors in phonon frequency are accountable for overestimated volumes and numerical instabilities based on these parameters, since $\Delta V/V_0$ characterizes contributions from thermally activated phonons only.

Considering that isothermal bulk modulus B_T is the second derivative of Helmholtz free energy F, *i.e.*, $B_T = V(\partial^2 F/\partial V^2)_T$, it could be very sensitive to noises in F, and a strong dependence on temperature was highlighted in the literature.[15,16] This offers an efficient criterion for evaluating the accuracy of thermal effects described by various basis sets and Hamiltonians. B_T of FI and FII are calculated numerically with three-point central differences around the equilibrium volume, and their temperature dependence are compared in **Figure S5** with the data derived from isothermal measurements by Boldyreva *et al.* at 295 K and variable pressures. The procedures for processing reference data have been introduced in **Section S2.3**.

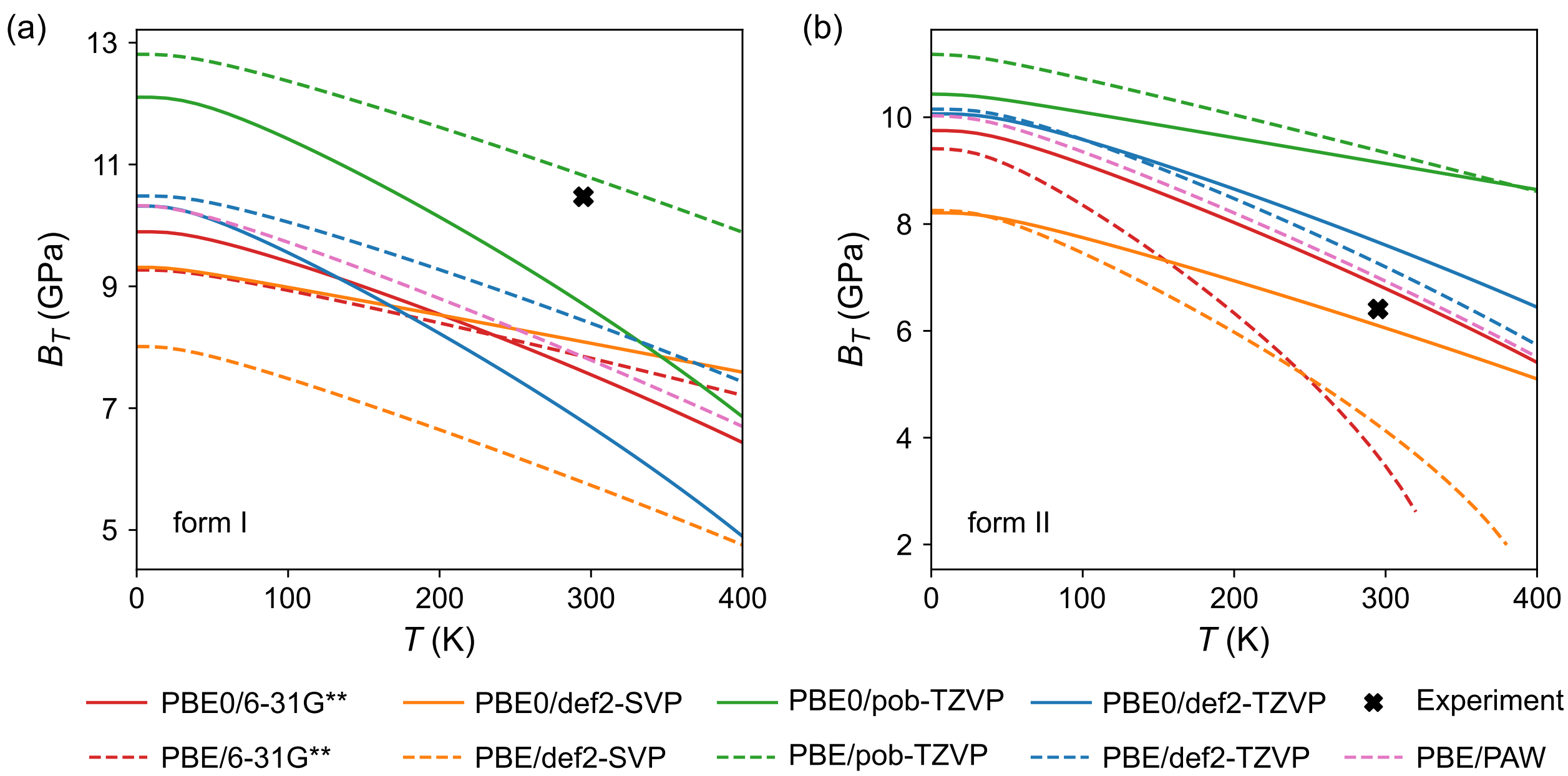

Figure S5: Temperature dependence of the bulk moduli B_T of (a) form I and (b) form II paracetamol.

Remarkable consistency is noticed among the $T - B_T$ curves predicted by PBE/def2-TZVP, PBE0/6-31G** and PBE/PAW, whereas PBE0/def2-TZVP yields comparable results

for FII. The outlier of PBE0/def2-TZVP for FI corresponds to the overestimated volumes and probably indicates that the predicted free energy derivatives are consistent with PBE/def2-TZVP and PBE/PAW. In comparison, given the overestimated volumes, the rather surprising agreement achieved with PBE0/6-31G** probably benefits from error cancellations, which is counterbalanced by the underestimated $(\partial^2 F/\partial V^2)_T$. Predictions based on other parameterizations deviate from the converging trend, suggesting that atomic basis sets smaller than def2-TZVP are generally insufficient to predict bulk moduli. Converged results based on PBE/def2-TZVP and PBE/PAW correctly reproduce the experimentally observed larger B_T of FI than that of FII, but the deviation from reference remains prominent for FI. Since def2-TZVP+gCP (PAW) is nearly (completely) free from basis set superposition errors (BSSE), the accuracy of Hamiltonian, instead of basis set, might be the underlying factor. Further alignment of the theoretical B_T with references probably requires a detailed comparison of various exchange-correlation functionals or dispersion correction schemes, which is beyond the scope of the current work.

S3.2 Linear expansions and lattice constants

Linear expansions along non-equivalent lattice constants of FI and FII are plotted in **Figure S6** with references. Double-ζ basis sets overestimate almost all axial lengths, indicating the nearly isotropic distribution of errors in volume predictions. For triple-ζ and plane-wave basis sets, the distribution of errors is more dispersed, with the most prominent differences in lattice vector b, along which the layered hydrogen bond networks are stacked (**Figure 1ab, manuscript**). Our previous work[2] has discussed the notable errors in b by pob-TZVP basis set for FI, despite its reasonable volume predictions. It is believed that both the large interlayer spacing in FI and the parameterization of gCP correction lead to this phenomenon, where gCP fails to fully account for the delicate London dispersion and $\pi - \pi$ stacking interactions that dominate along b. This necessitates the use of more diffuse basis sets, including def2-TZVP and plane-wave.

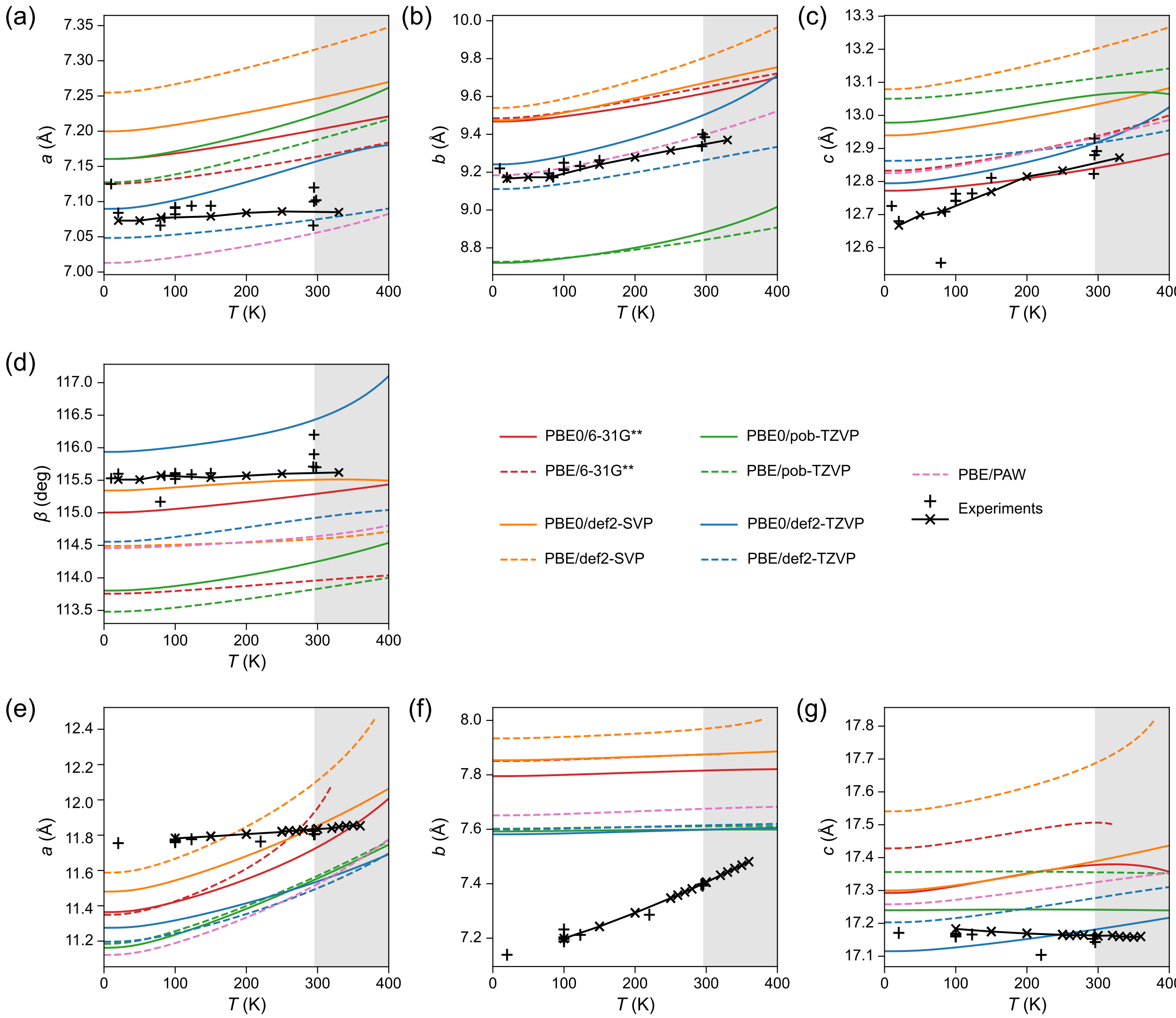

Figure S6: Temperature dependence of the non-equivalent lattice constants of (a)-(d) form I and (e)-(g) form II paracetamol. Symbols of corresponding lattice parameters are labeled along the y axes, which are consistent with the annotations in **Figure 1ab** of the manuscript. The gray-shaded areas are beyond the empirical validity limit of quasi-harmonic lattice dynamics.

However, results based on triple-ζ basis sets overall fail to reproduce the linear expansion trends of orthorhombic FII, despite the less number of symmetrically independent lattice parameters and (ideally) more stable fittings. This failure persists for the converged triple-ζ and plane-wave basis sets, and negligible improvements are made by incorporating Fock exchange. Considering that anisotropic energy minimization with respect to lattice parameters is circumvented in the proposed QHA LD scheme (**Equations 7–9, manuscript**),

it is necessary to discuss the potential improvements of adopting a fully anisotropic QHA LD scheme. In **Figure S7**, the linear and volumetric expansions obtained by PBE/PAW are compared with literature results based on PBE-MBD (many body dispersion correction) and the numerical atomic basis set, where free energy PES around the equilibrium lattice was perturbed using the second-order Taylor series.[17] To facilitate visual comparison, data are extracted from Figure S1 of the original publication with WebPlotDigitizer, and commensurate temperature ranges are set.

Performances of both methods are system-dependent. The present method yields better predictions for FI, probably suggesting the numerical instability of using fully anisotropic QHA LD for low-symmetry lattices. This can only be alleviated by increasing the number of volume points, which further escalates the already costly sampling. For example, perturbing the free energy PES of an orthorhombic lattice involves a non-linear fitting of ten unknowns, including equilibrium free energy, equilibrium lattice parameters, and a 3×3 Hessian matrix. In fact, to reduce the number of unknowns, the lattice parameter β of monoclinic FI was fixed in ref 17, which might further deteriorate the predictions. In contrast, the order of polynomials used by the present method to fit lattice parameters is tunable according to the number of available volume points, ensuring overdetermined fittings for both FI and FII, despite that only six volume points are sampled in this study.

For FII, the present method gives rise to significant errors in linear expansions, while the volumetric expansion agrees well with experimental references. This should be attributed to the '2-step' fitting scheme of the present method, where the equilibrium volume is obtained independent of lattice parameters. The failure of present method for linear expansions probably indicates that the thermal expansion of FII is more anisotropic than that of FI, necessitating fully anisotropic QHA LD methods. However, though the reference method achieves good agreement along the b and c axes, a qualitatively wrong thermal contraction was predicted along the a axis. The prominent error in a propagates to volume predictions, since the reference method depends purely on lattice parameters.

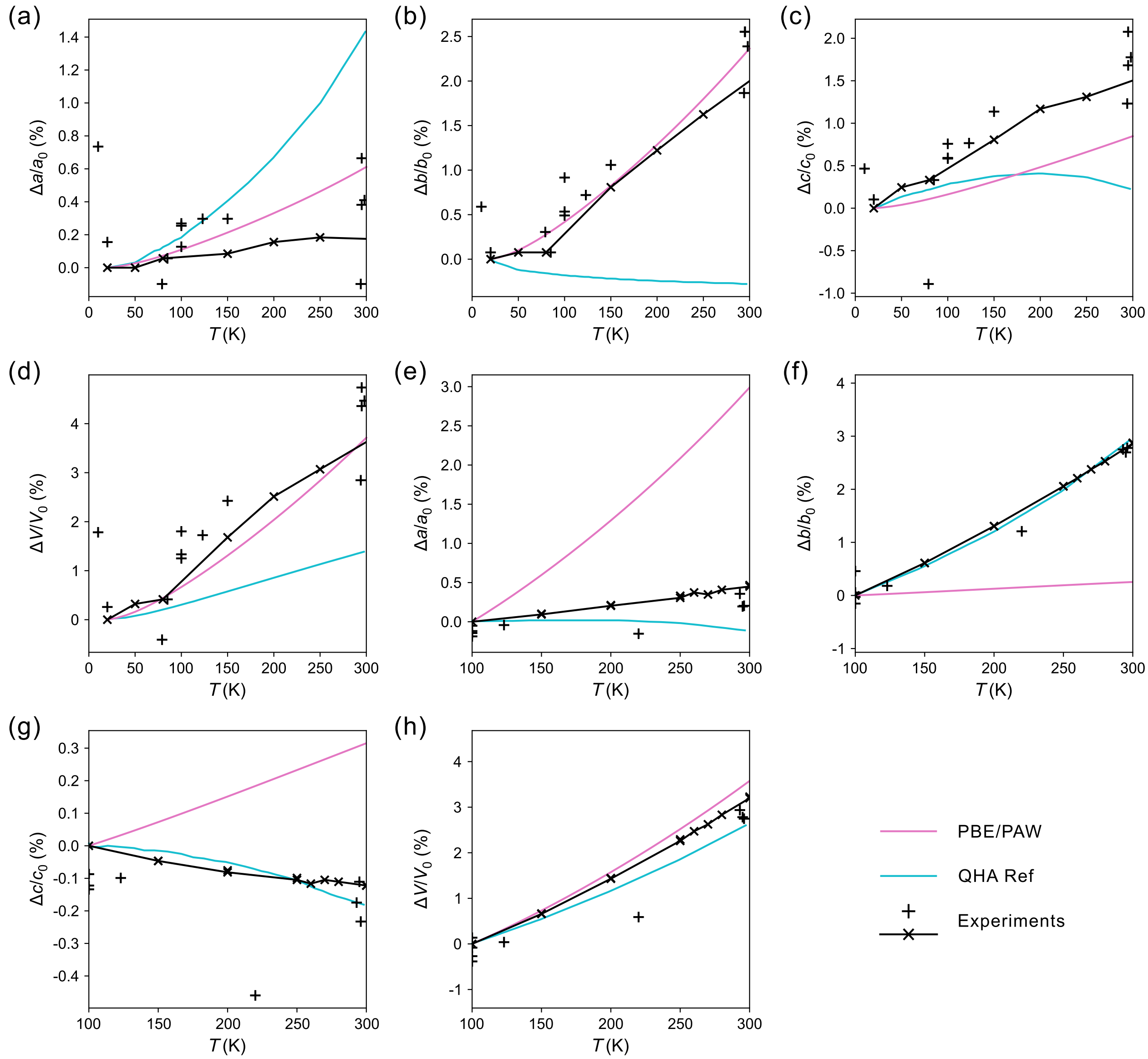

Figure S7: Linear (Δa, Δb, and Δc) and volumetric (ΔV) expansions as the percentage of low-temperature equilibrium values (a_0, b_0, c_0, and V_0) of (a)-(d) form I and (e)-(h) form II paracetamol, obtained with PBE/PAW. Due to the limit of available references, a_0, b_0, c_0, and V_0 are defined at 20 K (100 K) for form I (II). Symbols of corresponding lattice parameters are consistent with the annotations in **Figure 1ab** of the manuscript. The theoretical reference 'QHA Ref' is adopted with permission from ref 17. Copyright 2019 American Physical Society.

It is clear that both the proposed and the reference methods fail to satisfactorily predict the highly anisotropic expansion of FII. This is believed to originate from the absence of explicit expression of molecular configurations, or atomic coordinates, in the theoretical framework of QHA LD, which are discussed in detail in the following section. To incorpo-

rate temperature-dependent molecular configurations into the current theoretical framework without significantly increasing computational cost, a constrained-volume optimization is performed based on the finite-temperature volumes predicted by QHA LD. For completeness, the full set of results are listed in **Tables S6–S7** respectively for FI and FII, while corresponding discussions are detailed in the manuscript.

Table S6: Finite-temperature geometries of form I paracetamol[a]

**Hamiltonian**	**Basis Set**	**Volume (Å^3)**	***a*** **(Å)**	***b*** **(Å)**	***c*** **(Å)**	***β*** **(deg)**
PBE0	6-31G**	799.58 (4.67)	7.19 (1.40,-0.10)	9.57 (2.75,-0.12)	12.84 (0.06, 0.12)	115.11 (-0.42,-0.10)
	def2-SVP	818.24 (7.11)	7.23 (2.08,-0.04)	9.62 (3.27,-0.13)	13.03 (1.52, 0.11)	115.49 (-0.10,-0.00)
	pob-TZVP	758.20 (-0.75)	7.21 (1.76, 0.04)	8.84 (-5.15,-0.03)	13.04 (1.61,-0.07)	114.11 (-1.29,-0.02)
	def2-TZVP	778.79 (1.95)	7.14 (0.79,-0.01)	9.42 (1.10,-0.21)	12.90 (0.53, 0.11)	116.16 (0.49,-0.10)
PBE	6-31G**	811.56 (6.24)	7.15 (0.97,-0.01)	9.60 (3.08,-0.14)	12.92 (0.67, 0.05)	113.88 (-1.49,-0.04)
	def2-SVP	852.18 (11.56)	7.30 (3.05,-0.01)	9.72 (4.37,-0.21)	13.19 (2.79, 0.12)	114.50 (-0.95,-0.05)
	pob-TZVP	758.15 (-0.75)	7.18 (1.28, 0.03)	8.80 (-5.49,-0.14)	13.10 (2.12, 0.04)	113.69 (-1.65,-0.05)
	def2-TZVP	763.92 (0.00)	7.07 (-0.18, 0.07)	9.21 (-1.09,-0.20)	12.92 (0.65, 0.10)	114.83 (-0.67,-0.02)
	PAW	773.33 (1.23)	7.05 (-0.55, 0.02)	9.35 (0.39, 0.02)	12.92 (0.71, 0.10)	114.76 (-0.72, 0.15)
**Experiment**		763.9	7.086(2)	9.315(2)	12.833(3)	115.60(2)

[a] The environmental conditions, reference geometry, QHA data, and meanings of the numbers in parentheses are consistent with those presented in **Table 1, manuscript**.

Table S7: Finite-temperature geometries of form II paracetamol[a]

**Hamiltonian**	**Basis Set**	**Volume (Å^3)**	***a*** **(Å)**	***b*** **(Å)**	***c*** **(Å)**
PBE0	6-31G**	1593.10 (5.89)	11.88 (0.40, 1.29)	7.72 (4.29,-1.19)	17.36 (1.14,-0.12)
	def2-SVP	1622.88 (7.87)	11.96 (1.03, 0.89)	7.80 (5.27,-1.00)	17.41 (1.42, 0.09)
	pob-TZVP	1513.44 (0.60)	11.55 (-2.46,-0.08)	7.60 (2.66, 0.05)	17.24 (0.46,-0.00)
	def2-TZVP	1506.39 (0.13)	11.76 (-0.68, 1.90)	7.44 (0.52,-2.03)	17.21 (0.29, 0.17)
PBE	6-31G**	1647.24 (9.49)	11.85 (0.11,-0.86)	7.94 (7.20, 0.82)	17.51 (2.02, 0.01)
	def2-SVP	1706.92 (13.46)	12.07 (1.99,-0.30)	8.00 (7.96, 0.32)	17.68 (3.04,-0.04)
	pob-TZVP	1527.72 (1.55)	11.53 (-2.58,-0.33)	7.63 (3.08, 0.30)	17.36 (1.12,-0.00)
	def2-TZVP	1510.85 (0.43)	11.67 (-1.39, 1.51)	7.48 (0.95,-1.82)	17.31 (0.88, 0.20)
	PAW	1532.43 (1.86)	11.66 (-1.48, 1.21)	7.58 (2.39,-1.21)	17.33 (0.97, 0.01)
**Experiment**		1504.4(7)	11.837(7)	7.4057(13)	17.162(3)

[a] The environmental conditions, reference geometry, QHA data, and meanings of the numbers in parentheses are consistent with those presented in **Table 1, manuscript**.

S3.3 Variations in molecular configuration

Temperature-dependent variations in molecular configuration might disrupt the balance among packed molecules and alter lattice constants, depending on the specific geometry. However, in the current QHA scheme, thermal effects on molecular configuration can only be indirectly accounted for by the perturbed chemical environment within contracted or expanded lattices. As a compromise due to constrained computational resources, this method avoids optimizations on the high-dimensional free energy PES but remains insufficient for relatively large and soft molecules, leading to deviations from experiment as presented in the previous section.

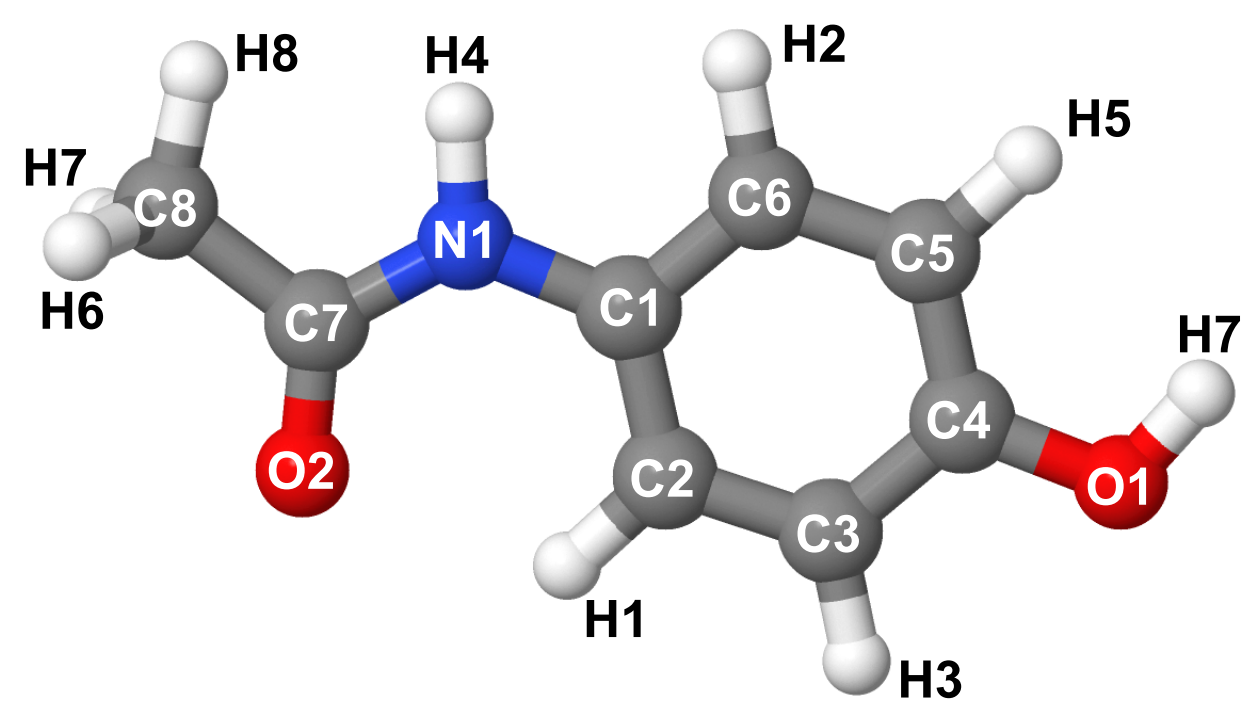

Figure S8: The C7-N1-C1-C2 torsion angle of paracetamol molecule. Atomic labels are consistent with ref 11

The variable-temperature measurement of FII by Drebushchak and Boldyreva[11] identified an increase in the C7-N1-C1-C2 torsion angle between hydroxyphenyl and acetamido groups of paracetamol molecule (**Figure S8**) as the temperature raises. This distortion reduces the length of molecular skeleton (O1 to C8), which has a large component along c and is perpendicular to b of FII lattice (**Figure 1b, manuscript**), requiring shrinkage in c and expansion in b to accommodate it.

To characterize how much the current QHA LD scheme can capture the delicate configurational freedom of paracetamol molecules within lattice, the C7-N1-C1-C2 torsion angle, which is optimized under athermal condition (ϕ_0), is compared with that optimized at finite-

temperature volume (ϕ_T, T = 250 K for FI, 300 K for FII) in **Table S8**. The difference between ϕ_T and ϕ_0, $\Delta\phi$, is adopted to describe the temperature-induced variations in molecular configurations. Data from consecutive variable-temperature measurements of FI[10] and FII[11] are adopted to reduce errors caused by inconsistent instrumental setups or refining methods. The temperatures of measurement are annotated in the subscripts of ϕ. The temperature ranges adopted for theoretical and experimental torsion angles, though discrepant due to limited reference available, are sufficient for qualitative analysis.

Table S8: The C7-N1-C1-C2 torsion angle of form I and form II paracetamol (Unit: deg)

**Polymorph**	**Hamiltonian**	**Basis Set**	ϕ_0	ϕ_T	$\Delta\phi$
form I $\phi_{20K} = 22.25$ $\phi_{250K} = 23.30$ $\Delta\phi = 1.05$	PBE0	6-31G**	21.65	21.54	-0.11
		def2-SVP	21.31	20.98	-0.33
		pob-TZVP	24.51	28.27	3.75
		def2-TZVP	20.99	21.58	0.58
	PBE	6-31G**	19.22	19.04	-0.18
		def2-SVP	19.16	19.21	0.06
		pob-TZVP	21.02	23.59	2.56
		def2-TZVP	18.64	19.12	0.48
		PAW	18.30	18.78	0.48
form II $\phi_{100K} = 16.70$ $\phi_{300K} = 18.93$ $\Delta\phi = 2.23$	PBE0	6-31G**	15.74	17.06	1.32
		def2-SVP	17.26	18.99	1.73
		pob-TZVP	19.35	21.97	2.63
		def2-TZVP	15.54	16.32	0.78
	PBE	6-31G**	14.77	15.57	0.83
		def2-SVP	16.48	18.13	1.64
		pob-TZVP	17.20	18.86	1.65
		def2-TZVP	14.86	15.55	0.70
		PAW	16.15	17.02	0.87

The influence of basis set is negligible, except for the outlier of pob-TZVP, whose results are deteriorated by serious BSSE, bringing difficulties for further investigation. Nevertheless, delicate influences of Hamiltonian are observed; including Fock exchange tends to slightly increase ϕ_0, ϕ_T and $\Delta\phi$, which distorts the molecule and shortens its skeleton. Literature results suggest enhanced non-covalent interactions between acetamido O2 and hydroxyphenyl H1,[18,19] leading to a 'ring structure' consisting of O2, H1 and the C7-N1-C1-C2 torsion angle. Fock exchange has been revealed to be critical for correctly capturing many-body interactions within hydrogen-bonded rings of similar structures.[20] Furthermore, despite the

general increase in distortion at higher temperatures, the estimated $\Delta\phi$ of FI and FII are lower than the references. This leads to more significant errors in FII, as it has larger $\Delta\phi$ and molecules consisting of its packing motif are better aligned along lattice vectors.

Error due to the insufficient description of molecular configuration can influence both the sampled volume points and the constrained optimizations at predicted volumes, since both steps are fundamentally based on the same athermal PES. Therefore, this error can propagate throughout the entire QHA LD workflow and is difficult to eliminate without explicitly expressing atomic coordinates in the formulation of free energy, which can significantly increase the dimensionality of optimization problems. This phenomenon is probably more prominent for molecular crystals, especially those consisting of soft molecules, while crystals bound by stronger interactions are expected to have limited configurational freedom and therefore are less influenced.

S3.4 Phonon continuity

For each phonon mode, a constant and mode-specific Grüneisen parameter γ is fitted to obtain its volume-dependent frequency $\omega(V)$ (**Equation 5, manuscript**) based on frequencies at sampled volume points. However, errors due to variable lattice, such as Pulay forces, can disrupt the original ordering of modes, necessitating the establishment of cross-volume phonon continuity prior to any meaningful fittings. Although the eigenvector-based algorithm given in **Equation 6, manuscript** is pertinent for this task in most cases, it might fail due to prominent variations in geometry or softening of phonon modes, increasing numerical noises in $\omega(V)$ and free energy PES. The coefficient of determination R^2 characterizes the quality of Grüneisen fittings and provides insight into the continuity of corresponding mode.

Figure S9 illustrates the distributions of R^2 based on various parameterizations. Basis set prominently affects the results, whereas differences due to the inclusion of Fock exchange are nearly negligible for triple-ζ basis sets, which are similar to observations of structural properties, indicating the crucial role of $\omega(V)$ in geometry prediction. As suggested by the

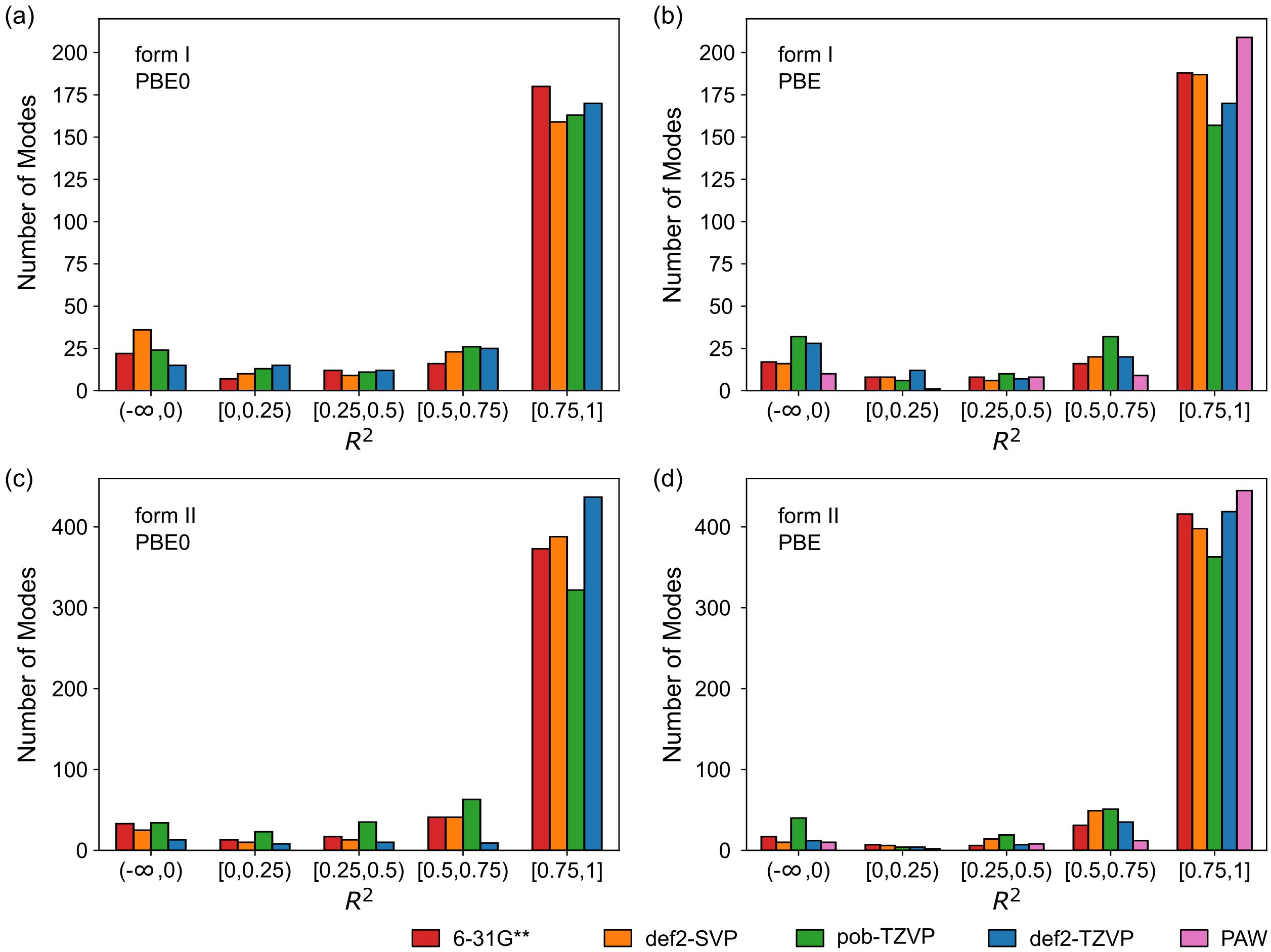

Figure S9: Distributions of the coefficient of determination (R^2) of the fitted mode-specific Grüneisen parameters.

largest number of modes with $R^2 \geq 0.75$, the plane-wave basis set achieves the optimal fitting quality and continuity throughout the volume range. Using atomic basis sets generally lead to larger errors, even with the def2-TZVP basis set that predicts comparable structural properties to plane-wave. Besides, although with harmonic LD, the predictions based on pob-TZVP for FII are analogous to those of def2-TZVP and plane-wave,[2] this conclusion is apparently not transferable to QHA LD, as indicated in **Figure S9cd**.

The failure of atomic basis sets probably indicates that QHA LD is more vulnerable to BSSE than harmonic LD, which should be attributed to how the gCP correction was parameterized. While gCP correction typically enhances the lattice binding energy profiles, it was originally developed for the athermal equilibrium lattice, and its error escalates in the

short and long-range.[21,22] Since QHA LD fittings require samplings at a series of volumes, this error can deteriorate the results with large deviations from the equilibrium, reducing phonon continuity across the sampled range.

Furthermore, despite being BSSE-free, poor fittings of $\omega(V)$ with $R^2 < 0$ are observed for the plane-wave basis set, probably indicating that the monotonic $\omega(V)$ as depicted by the Grüneisen model might be insufficient for certain phonon modes, which is a general problem for all parameterizations. This phenomenon is probably associated with the soft and highly anharmonic intermolecular vibrations, where the current theoretical framework and sampling method based on normalized phonon modes might be insufficient for accurate descriptions.[17,23] However, anharmonicities arising from higher-order terms and phonon-phonon coupling are beyond the scope of QHA LD workflows and the current work.

S3.5 Intermolecular vibrations

The distributions of phonon frequency of FI and FII can be unambiguously partitioned into three regions with distinct vibrational patterns:

- The low-frequency region (0∼250 cm^{-1}), where intermolecular vibrations dominate, involving collective motions of molecules in the lattice;
- The medium-frequency region (250∼1750 cm^{-1}), where intramolecular vibrations dominate, involving bending modes of all atoms and stretching modes of heavy atoms;
- The high-frequency region (2800∼3400 cm^{-1}), where intramolecular vibrations dominate, involving stretching modes of the hydrogen atom.

Low-frequency intermolecular vibrations between 0 and 250 cm^{-1} are associated with the delicate non-covalent binding between molecules, which yields critical information for polymorph discrimination.[5,17,24] Therefore, the performance of basis set and Hamiltonian in predicting phonons within this region have to be carefully examined to unambiguously identify the specific microscopic mechanism underlying polymorph transitions.

Apart from the data presented in the manuscript, **Figure S10** offers a more comprehensive comparison between phonon frequencies based on QHA LD (ν_{QHA}) and harmonic LD (ν_{HA}) in the low-frequency region. Atomic basis sets generally obtain results similar to the plane-wave basis set for FI, whereas the alignment between ν_{QHA} and ν_{HA} is reduced in the case of FII. As the largest atomic basis set tested, def2-TZVP shows closer proximity to smaller atomic basis sets in this scenario, rather than the plane-wave basis set, stressing the cruciality of basis set completeness. The origin of this phenomenon, as analyzed in the manuscript, lies in the Pulay forces induced by variable atomic coordinates, whose influences are more prominent for FII with larger distortions in molecular configurations.

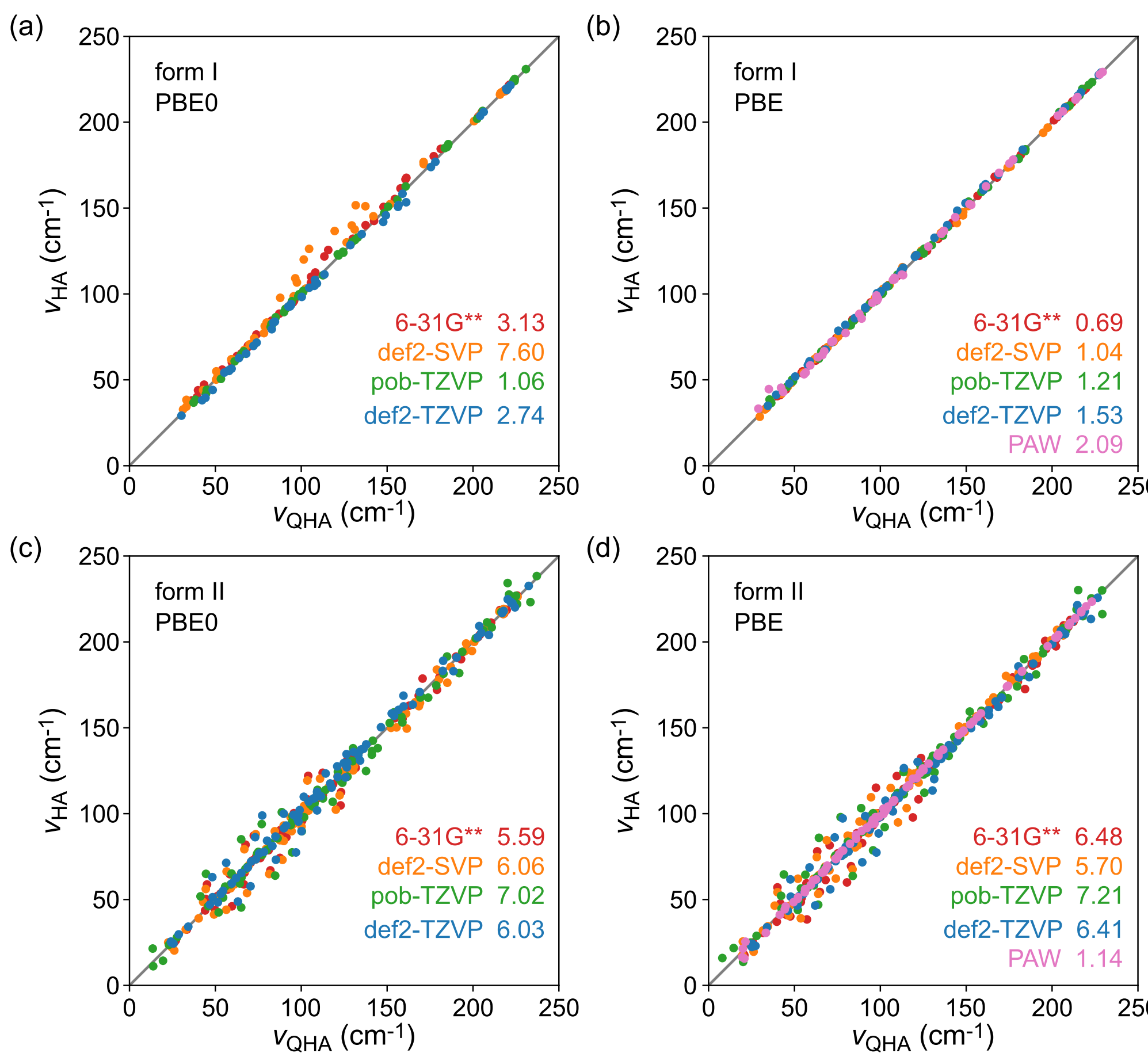

Figure S10: Harmonic intermolecular phonon frequencies ν_{HA} at the equilibrium volume of 300 K, as functions of quasi-harmonic phonon frequencies ν_{QHA} at the same volume. The polymorph and Hamiltonian are annotated on the upper left corner of each subplot. Other marks and annotations are consistent with those in **Figure 2ab** of the manuscript.

The trends observed in **Figures S9–S10** highlight the robustness of plane-wave basis set for QHA fittings, which successfully establishes the consistency between analytical results based on QHA LD and numerical results based on harmonic LD.

Figure S11 presents the low-frequency polycrystalline Raman spectra of FI and FII at 300 K, where all parameterizations tested in this work are compared. In all cases, errors are noticed for the peaks at ~80 cm^{-1} of FI and ~140 cm^{-1} of FII, where the corresponding modes exhibit strong anharmonicity due to rotations along the molecular skeleton.[2,17] The overestimated volumes of double-ζ basis sets weaken the intermolecular bindings and red-shift Raman peaks, whereas the inclusion of Fock exchange improves the agreement with experiments owing to the more compact lattice. Peaks based on the pob-TZVP basis set are

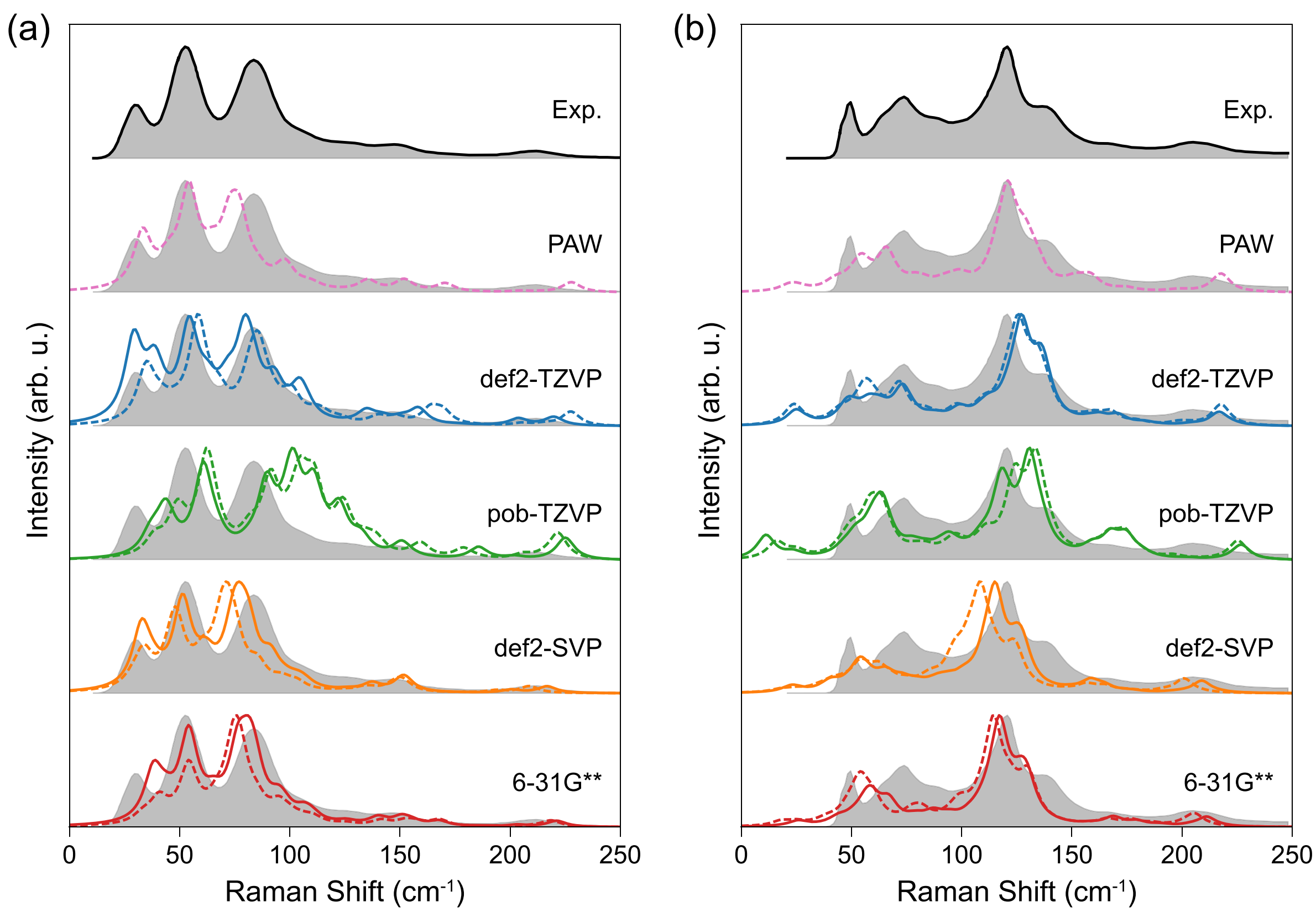

Figure S11: Experimental and calculated Raman spectra of (a) form I and (b) form II paracetamol between 0 and 250 cm^{-1} at the temperature of 300 K. Spectra in solid lines are obtained by PBE0, while those in dashed lines are obtained by PBE. Other marks and annotations are consistent with those in **Figure 2cd** of the manuscript. Experimental spectra adapted with permission from ref 5. Copyright 2012 American Chemical Society.

blue-shifted by ~20 cm^{-1} with prominent errors in their intensities, again highlighting the systematic failure of this basis set to be used for QHA LD.

S3.6 Intramolecular vibrations

Intramolecular vibrations beyond 250 cm^{-1} are dominated by covalent interactions localized within the molecule, so they are believed to exert limited influences on the macroscopic properties and transitions of the crystal. Considering the identical chemical formula of molecular polymorphs, it is also expected that contributions from intramolecular vibrations can largely counterbalance with each other when ranking their relative stabilities. Nevertheless, vibrational properties beyond 250 cm^{-1} are presented here with a brief analysis for the completeness of the results.

Table S9: Root-mean-square-deviation between quasi-harmonic and harmonic intramolecular frequencies at the the equilibrium volume of 300 K (Unit: cm^{-1})

**Polymorph**	**Hamiltonian**	**Basis Set**	**250~1750 cm^{-1}**	**2800~3400 cm^{-1}**
form I	PBE0	6-31G**	0.64	0.87
		def2-SVP	0.77	0.65
		pob-TZVP	5.30	1.56
		def2-TZVP	0.70	1.30
	PBE	6-31G**	0.65	1.86
		def2-SVP	1.17	1.86
		pob-TZVP	1.41	3.81
		def2-TZVP	0.73	1.65
		PAW	0.34	3.03
form II	PBE0	6-31G**	4.11	3.34
		def2-SVP	4.02	4.15
		pob-TZVP	3.82	3.26
		def2-TZVP	4.30	2.14
	PBE	6-31G**	4.00	3.03
		def2-SVP	3.61	2.28
		pob-TZVP	4.69	2.93
		def2-TZVP	3.88	4.56
		PAW	0.26	1.15

RMSD between ν_{QHA} and ν_{HA} in the medium-to-high-frequency regions are listed in **Table S9**, which are based on the same geometries at 300 K as in **Figure S10**. It should

be noted that all ν_{HA} are scaled by the factors listed in **Table S1** to match ν_{QHA}, which are fitted on scaled harmonic frequencies. The observed trend is consistent with what has been discussed for intermolecular modes, that RMSDs of atomic basis sets are generally larger for FII due to more significant variations in molecular configuration. Such variations can alter both intramolecular and intermolecular phonons, and the failure to account for their influences, as suggested by ν_{QHA}, leads to systematic deviations across the frequency range. Besides, the limited RMSD of PBE/PAW here again clarifies that Pulay forces originating from atomic coordinates, instead of lattice cells, are the dominant source of error influencing the accuracy of QHA LD for molecular crystals, especially those consisting of soft molecules.

The intramolecular Raman spectra of FI and FII at 300 K are plotted respectively in **Figures S12–S13**, which are based on the same harmonic LD calculation as presented just above. In addition to the frequency scaling factors listed in **Table S1**, phonon modes with prominent anharmonicity, including the stretching along O−H and N−H bonds, are scanned with the `ANHARM` keyword of C23 to account for the nuclear quantum effects of delocalized H atoms in hydrogen bonds.[25] The harmonic (ν_{ha}) and anharmonic frequencies (ν_{aha}) of these modes are reported in **Table S10**, and only the results based on atomic orbitals are reported due to technical issues.

The role of basis set becomes marginal in the medium-to-high-frequency region of Raman spectra, while including Fock exchange slightly blue-shifts the peaks beyond 1500 cm^{-1}, Remarkable differences are noticed in the high-frequency region, where peaks corresponding to O−H and N−H stretching modes are prominently red-shifted upon the inclusion of anharmonic nuclear quantum effects. This leads to large deviations from the references in the spectra based on PBE, whereas the PBE0-based spectra are better aligned. The Raman spectra presented here are almost identical to those obtained in our previous work,[2] which were simulated using athermal equilibrium geometries. Further discussion of potential sources of error are required to improve the agreement with experiment, where thermal effects can be excluded considering the nearly negligible influences of thermal expansion on intramolecular

Table S10: Frequencies of O−H and N−H stretching modes (Unit: cm^{-1})

**Polymorph**	**Hamiltonian**	**Basis Set**	ν_{ha}**(OH)**	ν_{aha}**(OH)**	ν_{ha}**(NH)**	ν_{aha}**(NH)**
form I	PBE0	6-31G**	3384	3043	3507	3334
		def2-SVP	3353	3019	3477	3303
		pob-TZVP	3307	2949	3456	3266
		def2-TZVP	3359	3014	3500	3328
	PBE	6-31G**	3070	2653	3344	3157
		def2-SVP	3079	2699	3316	3137
		pob-TZVP	3019	2594	3283	3073
		def2-TZVP	3019	2592	3315	3114
form II	PBE0	6-31G**	3409	3090	3488	3309
		def2-SVP	3365	3047	3464	3282
		pob-TZVP	3238	2856	3414	3201
		def2-TZVP	3365	3001	3464	3299
	PBE	6-31G**	3125	2745	3334	3147
		def2-SVP	3102	2743	3311	3129
		pob-TZVP	2953	2522	3262	3033
		def2-TZVP	3023	2612	3321	3116

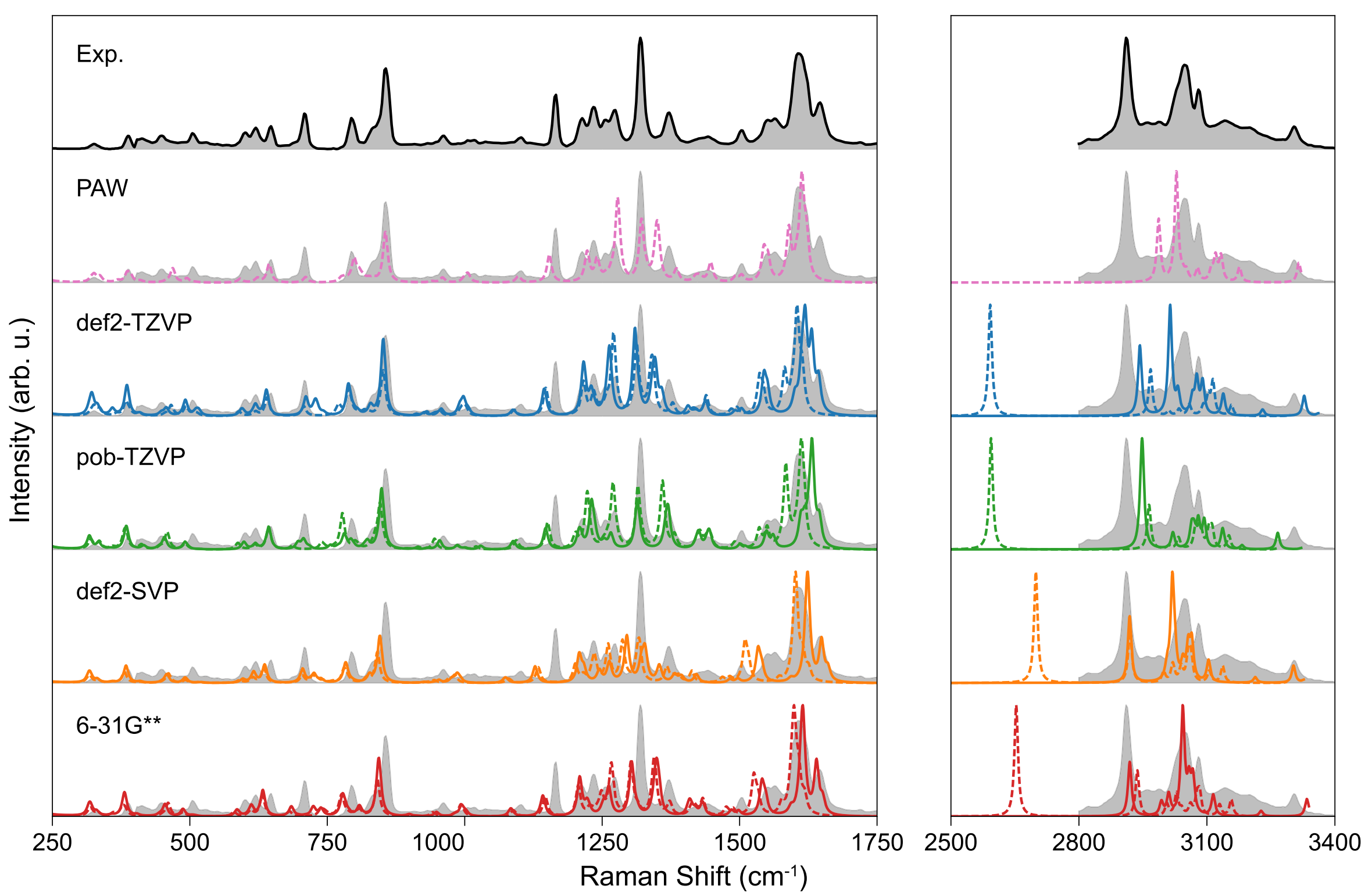

Figure S12: The calculated Raman spectra of form I paracetamol (left) from 250 to 1750 cm^{-1} and (right) from 2500 to 3400 cm^{-1} at the temperature of 300 K. Other marks and annotations are consistent with those in **Figure S11**. Experimental spectra adapted with permission from ref 5. Copyright 2012 American Chemical Society.

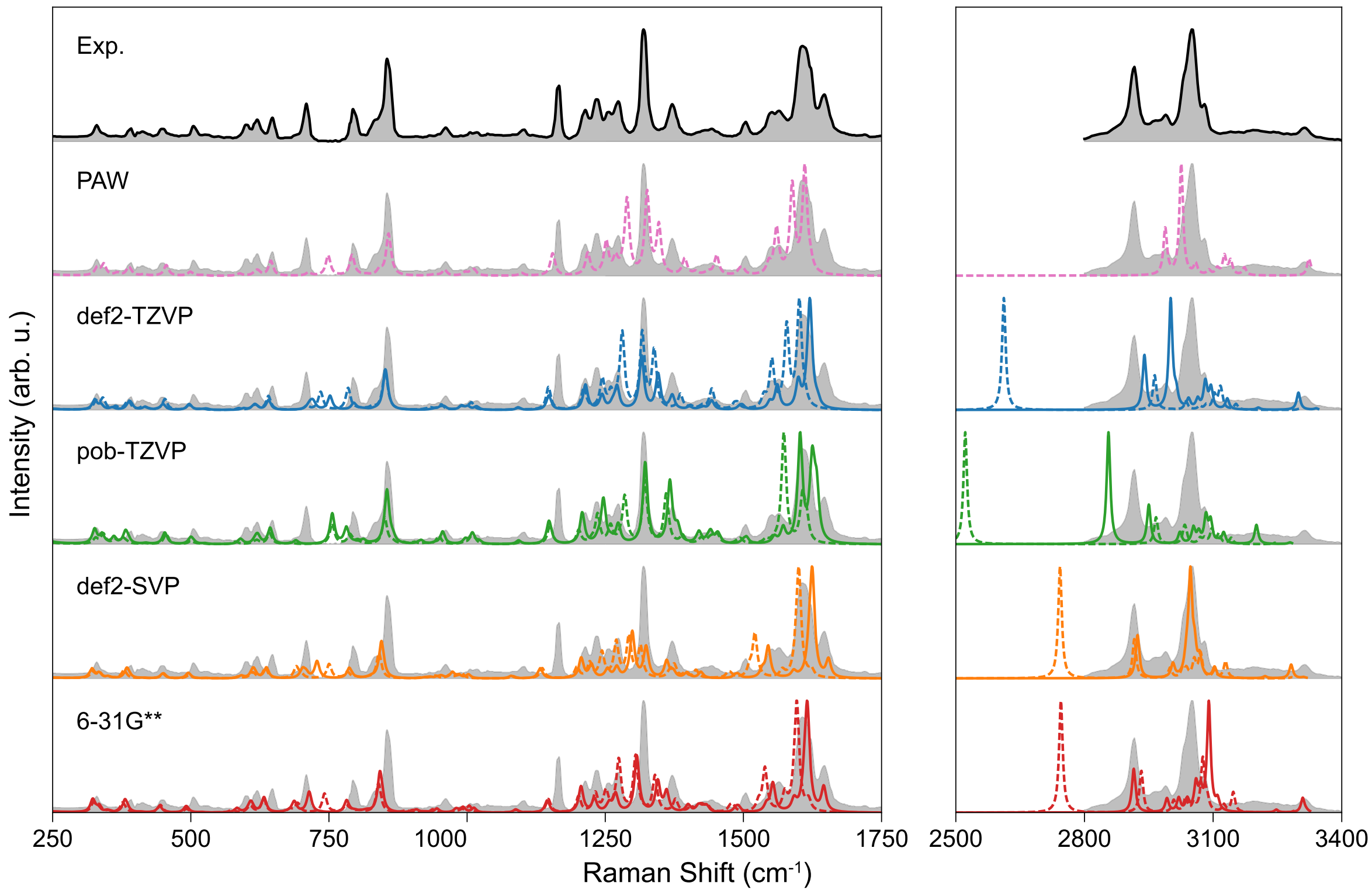

Figure S13: The calculated Raman spectra of form II paracetamol (left) from 250 to 1750 cm^{-1} and (right) from 2500 to 3400 cm^{-1} at the temperature of 300 K. Other marks and annotations are consistent with those in **Figure S11**. Experimental spectra adapted with permission from ref 5. Copyright 2012 American Chemical Society.

vibrations, as revealed in this study. Alternatively, the potential scope can be narrowed to the description of more localized interactions, such as distortions in the molecular skeleton or many-body interactions within the hydrogen-bonded chain of NH. . . OH. . . O=C.

S3.7 Thermochemical properties

The complete set of QHA-derived thermochemical properties is collected under standard conditions (298.15 K, 101.3 kPa) for FI and FII in **Tables S11–S12**, respectively, which includes the constant-pressure specific heat C_p°, and the Gibbs free energy ΔG_{sub}°, enthalpy ΔH_{sub}° and entropy ΔS_{sub}° of sublimation. Since the dominant sources of error underpinning these properties have been clearly elucidated in the manuscript, here we first present the supplementary analysis of smaller atomic basis sets. The errors in ΔS_{sub}° are then discussed

in detail.

Results of C_p° generally agree well with references, except that those predicted by PBE/6-31G** and PBE/def2-SVP for FII are overestimated by more than 10 J mol^{-1} K^{-1}. This further supports the conclusion that C_p° is vulnerable to noise on free energy PES, given the severe numerical instabilities of these parameterizations (**Figure S3**). For ΔG_{sub}°, predictions obtained by triple-ζ and plane-wave basis sets are generally higher than those of double-ζ basis sets, which are believed to originate from stronger intermolecular bindings within more compact lattices. The inclusion of Fock exchange also reduces ΔG_{sub}°, similar to the trend presented in the manuscript. However, this effect becomes less prominent for double-ζ basis sets, probably due to the counterbalancing effects of PBE0-based structures, where the more compact lattices lead to stronger bindings and therefore to higher ΔG_{sub}°.

The overexpansion obtained with double-ζ basis sets also reduces ΔH_{sub}° by up to 20 kJ mol^{-1}, which improves the agreement with values documented in refs. 26,27. However, according to the theoretical benchmarking work by McKinley and Beran,[28] concerns were raised regarding the accuracy of corresponding sublimation calorimetry experiments, since a systematic error around 12 kJ mol^{-1} persists between the reported ΔH_{sub}° of FI and those from other sources. This could reduce the reliability of ΔH_{sub}° of FII and ΔS_{sub}° of both phases reported in the same references, since they are derived using ΔH_{sub}° and ΔG_{sub}° of FI, as well as the polymorphic transition enthalpy.

However, beyond the non-uniformly distributed 10∼50 J mol^{-1} K^{-1} deviations from references, a more significant issue of theoretical data emerges in the relative difference of ΔS_{sub}° between FI and FII. All parameterizations tested in this study predict higher ΔS_{sub}° for FI, indicating that FI would exhibit lower vibrational entropy than FII does under standard conditions. In contrast, the opposite has been independently verified in two experiments.[27,29]

This phenomenon should be attributed to the synergy between the free-rotating methyl group and the distinct packing motifs of paracetamol molecules in FI and FII. The corrugated hydrogen-bonded network of FI (**Figure 1a, manuscript**) increases interlayer distances,

Table S11: Thermochemical properties of form I paracetamol at 298.15 K, 101.3 kPa

**Hamiltonian**	**Basis Set**	C_p° **(J mol^{-1}K^{-1})**	ΔG_{sub}° **(kJ mol^{-1})**	ΔH_{sub}° **(kJ mol^{-1})**	ΔS_{sub}° **(J mol^{-1}K^{-1})**
PBE0	6-31G**	182.63	55.43	118.23	210.64
	def2-SVP	184.45	55.48	121.62	221.84
	pob-TZVP	183.92	65.53	125.84	202.30
	def2-TZVP	188.99	57.22	126.05	230.88
PBE	6-31G**	183.73	58.39	125.90	226.43
	def2-SVP	188.90	54.48	119.67	218.66
	pob-TZVP	181.58	65.33	137.69	242.70
	def2-TZVP	179.55	66.92	135.14	228.81
	PAW	183.80	60.50	130.13	233.52
**Experiment**[a]		163±1<br>189.68	60.0	117.9±0.7<br>129.9±1.4	190±2
**Theory**[a]			58.8~80.7	131.0~154.1	242.1~246.3

[a] Reference data from the same sources as those presented in **Table 2, manuscript**.

Table S12: Thermochemical properties of form II paracetamol at 298.15 K, 101.3 kPa

**Hamiltonian**	**Basis Set**	C_p° **(J mol^{-1}K^{-1})**	ΔG_{sub}° **(kJ mol^{-1})**	ΔH_{sub}° **(kJ mol^{-1})**	ΔS_{sub}° **(J mol^{-1}K^{-1})**
PBE0	6-31G**	191.15	53.48	114.02	203.06
	def2-SVP	189.15	51.38	116.64	218.91
	pob-TZVP	186.34	67.15	123.78	189.96
	def2-TZVP	184.43	58.07	125.55	226.35
PBE	6-31G**	208.39	57.03	120.71	213.59
	def2-SVP	201.24	51.67	114.19	209.71
	pob-TZVP	187.27	68.03	136.25	228.82
	def2-TZVP	186.04	67.95	134.16	222.08
	PAW	189.33	62.41	129.39	224.65
**Experiment**[a]		191.5	56.1	115.9±0.9	200±3

[a] Reference data from the same sources as those presented in **Table 2, manuscript**.

which reduces steric effects and lowers the potential barrier of the rotating methyl group in FI, leading to quasi-free rotations at room temperature. Such rotations have been investigated in detail by calculations based on curvilinear coordinates.[23,29] In comparison, the Cartesian displacements adopted for finite-displacement LD might be insufficient to fully account for the amplitude of such rotations. As an inherent drawback of harmonic/QHA LD, this consequently underestimates the entropy of FI and FII (*i.e.*, overestimates ΔS°_{sub}) and reverses their relative differences, since FI is more vulnerable in this scenario. Furthermore, this error smears the delicate energy differences between FI and FII, leading to significant deviations in predicted phase boundaries. Fully addressing this error probably necessitates the implementation of novel displacement sampling methods that can offer a detailed free energy profile of molecular crystals as the function of intramolecular curvilinear motions. The study directly dedicated to this topic remains absent in the literature, as far as the authors are aware of. Nevertheless, by switching from harmonic LD to path-integral molecular dynamics (MD), where the relative stability of paracetamol polymorphs under ambient conditions was correctly reproduced, the energy difference between FI and FII was reported to decrease by over 3 kJ mol^{-1}.[30] Although this outcome might originate from multiple factors beyond the current scope, MD-related techniques are expected to grant higher rotational freedom for the methyl group, considering that they rely on ergodic sampling of configurational space instead of finite Cartesian displacements.

The complex interplay among the aforementioned factors, including the choice of basis set, Hamiltonian, and inherent errors of QHA LD, significantly escalates the difficulties of accurate polymorph rankings, since delicate differences in free energy can easily be smeared by error cancellations of counterbalancing thermal effects. To characterize the contributions of various thermodynamic functions to the relative stability of polymorphs, the difference in Helmholtz free energy ΔF between FI and FII at 300 K is partitioned into the differences of static internal energy ΔU_{DFT}, zero point energy ΔU_0, thermal vibrational energy ΔU_{vib}, and entropic term $-\Delta TS$, which are illustrated in **Figure S14**.

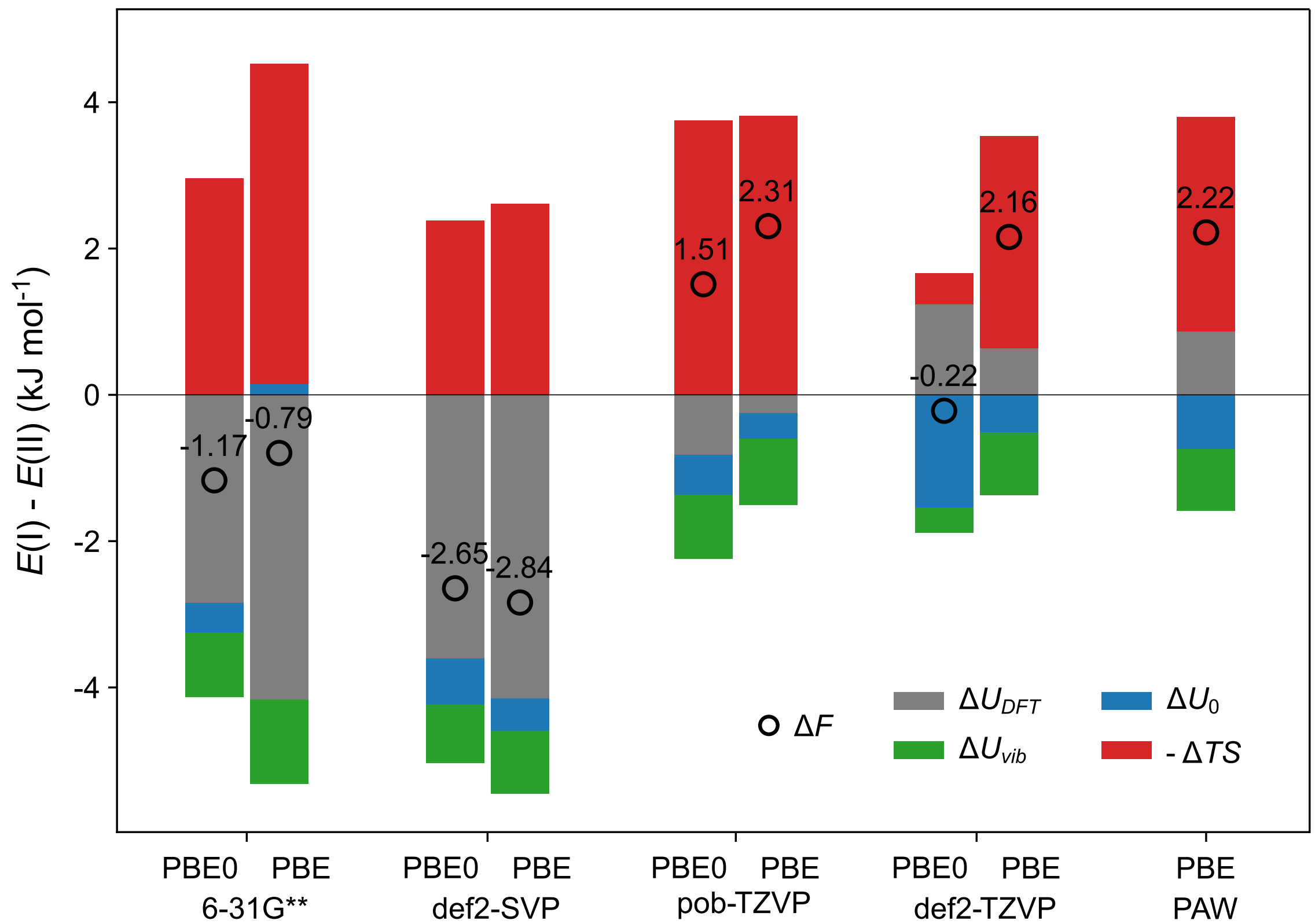

Figure S14: Differences in Helmholtz free energy ΔF between form I and form II paracetamol at 300 K. ΔF is partitioned respectively into the differences of static internal energy ΔU_{DFT}, zero-point energy ΔU_0, thermal vibrational energy ΔU_{vib}, and entropic term $-\Delta TS$. All the values are reported in kJ mol^{-1} per formula unit.

ΔU_{DFT} becomes increasingly positive and stabilizes FII over FI as the size of basis set increases, similar to the trend observed with the athermal equilibrium geometries.[2] This somewhat unexpected finding disproves the assumption in previous work that accounting for thermal expansion could reduce ΔU_{DFT}, since more prominent thermal expansions are expected (and obtained here) for FII to generate larger decreases in binding energy. In contrast, probably due to the weak and non-covalent nature of intermolecular bindings, the subtle difference in expansion rates is insufficient to induce appreciable changes in ΔU_{DFT}. Similarly, positive ΔU_{DFT} has also been previously reported using PBE-D3/TZV2P and harmonic LD,[30] indicating the ubiquitousness of this phenomenon. Further benchmarking of other exchange-correlation functionals, as well as dispersion correction methods, is probably needed before it can clearly conclude whether this observation originates from the inherent

artifact of PBE(PBE0)-D3 Hamiltonians, or it is a reliable depiction of the internal energy difference between FI and II.

For phonon contributions, the entropic term $-\Delta TS$ artificially stabilizes FII over FI as analyzed before, while phonon contributions to internal energy (ΔU_0 and ΔU_{vib}) destabilize FII, leading to the relative stability in reality. ΔU_0 and ΔU_{vib} counterbalance ΔU_{DFT}, which explains the correct relative differences of ΔH°_{sub} in **Tables S11–S12**. However, in almost all cases, $-\Delta TS$ dominates the symbol of ΔF, except for PBE0/def2-TZVP due to the overestimated volume of FI, which allows for higher entropy and reduces the difference. Although rotational modes are usually considered localized within the molecule, the high $-\Delta TS$ suggests that polymorph energy rankings cannot rely on fortuitous error cancellation between two phases, since the varied packing motifs might significantly influence the freedom and amplitude of rotations.[29] This again necessitates more advanced sampling techniques beyond the Cartesian displacements currently adopted by harmonic/quasi-harmonic LD to fully account for the peculiar thermodynamic properties of molecular crystals.

S3.8 The composite workflow

Supplementary results based on the composite workflow proposed in the manuscript are presented in this section for reference. The volume sampling and fitting steps of quasi-harmonic LD are performed at the PBE/PAW level to obtain the equilibrium volumes of FI and FII at 300 K, whereas the following constant-volume geometry optimization and harmonic LD calculations are performed with PBE0/def2-TZVP. The geometrical, vibrational, and thermochemical properties obtained are summarized in **Table S13**, where the adopted symbols have the same meanings as previously introduced. Due to limited computational resources, the thermochemical properties of crystalline phases under standard conditions, including H° and S°, are approximated using **Equation S4**.

$$
\begin{aligned}
H^\circ &\approx H + C_p(298.15\text{K} - T) + (101.3\text{kPa} - p)(1 - \alpha T)V \\
S^\circ &\approx S + C_p \ln\left(\frac{298.15\text{K}}{T}\right) - (101.3\text{kPa} - p)\alpha V
\end{aligned}
\tag{S4}
$$

where H and S are derived from harmonic phonons based on PBE0/def2-TZVP at 300 K, while V, C_p and α are fitted with QHA LD based on PBE/PAW at the same temperature. p is in kiloPascal (kPa) and T is in Kelvin (K).

Table S13: Properties of form I and form II paracetamol by the workflow

**Polymorph**	form I	form II
$\boldsymbol{a}$ **(Å)**	7.15	11.78
$\boldsymbol{b}$ **(Å)**	9.42	7.55
$\boldsymbol{c}$ **(Å)**	12.90	17.23
$\boldsymbol{\beta}$ **(deg)**	116.13	
$\boldsymbol{\nu_{ha}}$**(OH) (cm^{-1})**	3351	3342
$\boldsymbol{\nu_{aha}}$**(OH) (cm^{-1})**	3002	3005
$\boldsymbol{\nu_{ha}}$**(NH) (cm^{-1})**	3497	3485
$\boldsymbol{\nu_{aha}}$**(NH) (cm^{-1})**	3322	3305
$\boldsymbol{\Delta G^\circ_{sub}}$ **(kJ mol^{-1})**	56.01	57.15
$\boldsymbol{\Delta H^\circ_{sub}}$ **(kJ mol^{-1})**	125.31	124.38
$\boldsymbol{\Delta S^\circ_{sub}}$ **(J mol^{-1} K^{-1})**	232.44	225.51

With the same workflow, the intramolecular Raman spectra of FI and FII at 300 K are presented in **Figure S15**. The frequency scaling factor for PBE0/def2-TZVP (**Table S1**) is adopted to uniformly scale the spectra beyond 250 cm^{-1}. Furthermore, the harmonic frequencies of O−H and N−H stretching modes ν_{ha} are substituted by their anharmonic counterparts ν_{aha} to account for the nuclear quantum effects (**Table S13**).

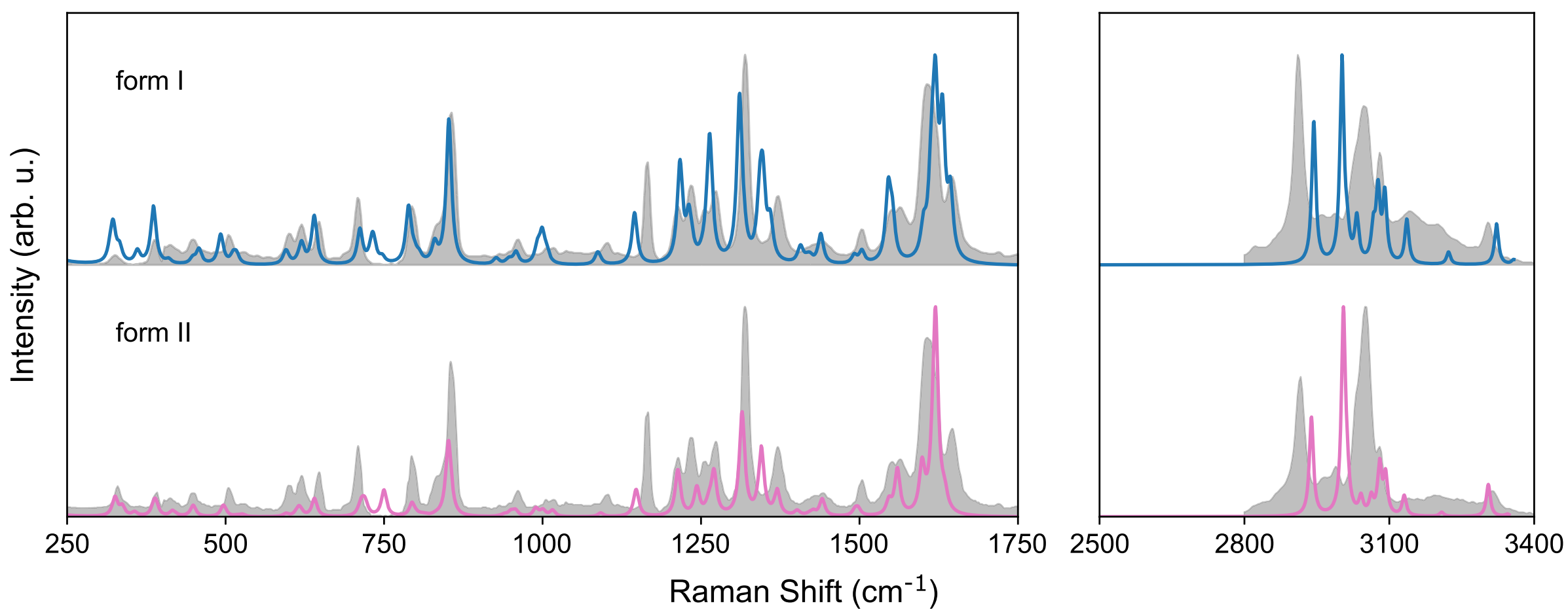

Figure S15: The calculated Raman spectra of form I and form II paracetamol (left) from 250 to 1750 cm^{-1} and (right) from 2500 to 3400 cm^{-1} at the temperature of 300 K by the workflow. The corresponding polymorph is annotated on the upper left corner of each subplot. For comparison, the experimental references are replicated as gray-shaded areas in the background, and the calculated intensities are normalized to the highest peak of each plot. Experimental spectra adapted with permission from ref 5. Copyright 2012 American Chemical Society.

References

(1) Dal Corso, A. Pseudopotentials periodic table: From H to Pu. *Computational Materials Science* **2014**, *95*, 337–350.

(2) Zhou, H.; Mallia, G.; Harrison, N. M. Influences of Basis Set and Electronic Exchange-Correlation on Low-Frequency Vibrations and Stability of Paracetamol Polymorphs. *Journal of Chemical Theory and Computation* **2025**, *21*, 9832–9843.

(3) Seko, A.; Togo, A. Projector-based efficient estimation of force constants. *Physical Review B* **2024**, *110*, 214302.

(4) Grimme, S. Supramolecular Binding Thermodynamics by DispersionCorrected Density Functional Theory. *Chemistry - A European Journal* **2012**, *18*, 9955–9964.

(5) Nanubolu, J. B.; Burley, J. C. Investigating the Recrystallization Behavior of Amorphous Paracetamol by Variable Temperature Raman Studies and Surface Raman Mapping. *Molecular Pharmaceutics* **2012**, *9*, 1544–1558.

(6) Becket, G.; Beech-Brandt, J.; Leach, K.; Simpson, Z. P. A.; Turner, A.; Whiting, A. ARCHER2 Service Description. *Zenodo* **2024**,

(7) Groom, C. R.; Bruno, I. J.; Lightfoot, M. P.; Ward, S. C. The Cambridge Structural Database. *Acta Crystallographica Section B: Structural Science, Crystal Engineering and Materials* **2016**, *72*, 171–179.

(8) IUCr *International Tables for Crystallography*, 5th ed.; Springer: Dordrecht, Netherlands, 2006; Vol. A: Space-Group Symmetry; Chapter 4.3. Symbols for space groups, pp 62–76.

(9) Stokes, H. T.; Hatch, D. M. FINDSYM: program for identifying the space-group symmetry of a crystal. *Applied Crystallography* **2005**, *38*, 237–238.

(10) Wilson, C. Variable temperature study of the crystal structure of paracetamol (p-hydroxyacetanilide), by single crystal neutron diffraction. *Zeitschrift für Kristallographie - Crystalline Materials* **2000**, *215*, 693–701.

(11) Drebushchak, T. N.; Boldyreva, E. V. Variable temperature (100-360 K) single-crystal X-ray diffraction study of the orthorhombic polymorph of paracetamol (p-hydroxyacetanilide). *Zeitschrift für Kristallographie - Crystalline Materials* **2004**, *219*, 506–512.

(12) Boldyreva, E. V.; Shakhtshneider, T. P.; Ahsbahs, H.; Sowa, H.; Uchtmann, H. Effect of High Pressure on the Polymorphs of Paracetamol. *Journal of Thermal Analysis and Calorimetry* **2002**, *68*, 437–452.

(13) Rohatgi, A. WebPlotDigitizer v5.2. 2025; `https://automeris.io`.

(14) Otero-de-la Roza, A.; Luaña, V. Gibbs2: A new version of the quasi-harmonic model code. I. Robust treatment of the static data. *Computer Physics Communications* **2011**, *182*, 1708–1720.

(15) Skelton, J. M.; Tiana, D.; Parker, S. C.; Togo, A.; Tanaka, I.; Walsh, A. Influence of the exchange-correlation functional on the quasi-harmonic lattice dynamics of II-VI semiconductors. *The Journal of Chemical Physics* **2015**, *143*, 064710.

(16) Skelton, J. M.; Parker, S. C.; Togo, A.; Tanaka, I.; Walsh, A. Thermal physics of the lead chalcogenides PbS, PbSe, and PbTe from first principles. *Physical Review B* **2014**, *89*, 205203.

(17) Raimbault, N.; Athavale, V.; Rossi, M. Anharmonic effects in the low-frequency vibrational modes of aspirin and paracetamol crystals. *Physical Review Materials* **2019**, *3*, 053605.

(18) Zhou, H.; Mallia, G.; Harrison, N. M. Ab initio simulation of molecular crystal regrowth of paracetamol from solution. *Materials Horizons* **2025**, *12*, 5162–5174.

(19) Mohammadi, M.; Khanmohammadi, A. Theoretical investigation on the non-covalent interactions of acetaminophen complex in different solvents: study of the enhancing effect of the cation- interaction on the intramolecular hydrogen bond. *Theoretical Chemistry Accounts* **2020**, *139*, 141.

(20) Howard, J. C.; Enyard, J. D.; Tschumper, G. S. Assessing the accuracy of some popular DFT methods for computing harmonic vibrational frequencies of water clusters. *The Journal of Chemical Physics* **2015**, *143*, 214103.

(21) Brandenburg, J. G.; Alessio, M.; Civalleri, B.; Peintinger, M. F.; Bredow, T.; Grimme, S. Geometrical Correction for the Inter- and Intramolecular Basis Set Superposition Error in Periodic Density Functional Theory Calculations. *The Journal of Physical Chemistry A* **2013**, *117*, 9282–9292.

(22) Sure, R.; Brandenburg, J. G.; Grimme, S. Small Atomic Orbital Basis Set FirstPrinciples Quantum Chemical Methods for Large Molecular and Periodic Systems: A Critical Analysis of Error Sources. *ChemistryOpen* **2016**, *5*, 94–109.

(23) Kapil, V.; Engel, E.; Rossi, M.; Ceriotti, M. Assessment of Approximate Methods for Anharmonic Free Energies. *Journal of Chemical Theory and Computation* **2019**, *15*, 5845–5857.

(24) Roy, S.; Chamberlin, B.; Matzger, A. J. Polymorph Discrimination Using Low Wavenumber Raman Spectroscopy. *Organic Process Research & Development* **2013**, *17*, 976–980.

(25) Pascale, F.; Tosoni, S.; Zicovich-Wilson, C.; Ugliengo, P.; Orlando, R.; Dovesi, R. Vibrational spectrum of brucite, Mg(OH)2: a periodic ab initio quantum mechanical calculation including OH anharmonicity. *Chemical Physics Letters* **2004**, *396*, 308–315.

(26) Perlovich, G. L.; Volkova, T. V.; BauerBrandl, A. Towards an understanding of the molecular mechanism of solvation of drug molecules: A thermodynamic approach by crystal lattice energy, sublimation, and solubility exemplified by paracetamol, acetanilide, and phenacetin. *Journal of Pharmaceutical Sciences* **2006**, *95*, 2158–2169.

(27) Perlovich, G. L.; Volkova, T. V.; Bauer-Brandl, A. Polymorphism of paracetamol: Relative stability of the monoclinic and orthorhombic phase revisited by sublimation and solution calorimetry. *Journal of Thermal Analysis and Calorimetry* **2007**, *89*, 767–774.

(28) McKinley, J. L.; Beran, G. J. O. Identifying pragmatic quasi-harmonic electronic structure approaches for modeling molecular crystal thermal expansion. *Faraday Discussions* **2018**, *211*, 181–207.

(29) Tsapatsaris, N.; Kolesov, B. A.; Fischer, J.; Boldyreva, E. V.; Daemen, L.; Eckert, J.; Bordallo, H. N. Polymorphism of Paracetamol: A New Understanding of Molecular Flexibility through Local Methyl Dynamics. *Molecular Pharmaceutics* **2014**, *11*, 1032–1041.

(30) Rossi, M.; Gasparotto, P.; Ceriotti, M. Anharmonic and Quantum Fluctuations in Molecular Crystals: A First-Principles Study of the Stability of Paracetamol. *Physical Review Letters* **2016**, *117*, 115702.
